# Outcome trends and safety measures after 30 years of laparoscopic cholecystectomy: a systematic review and pooled data analysis

**DOI:** 10.1007/s00464-017-5974-2

**Published:** 2018-03-19

**Authors:** Philip H. Pucher, L. Michael Brunt, Neil Davies, Ali Linsk, Amani Munshi, H. Alejandro Rodriguez, Abe Fingerhut, Robert D. Fanelli, Horacio Asbun, Rajesh Aggarwal, on behalf of the SAGES Safe Cholecystectomy Task Force

**Affiliations:** 1 Department of Surgery and Cancer, Imperial College London, London, UK; 2 Section of Minimally Invasive Surgery, Department of Surgery, Washington University School of Medicine, St. Louis, MO USA; 3 MRC Integrative Epidemiology Unit, School of Social and Community Medicine, University of Bristol, Bristol, UK; 4 School of Social and Community Medicine, University of Bristol, Barley House, Oakfield Grove, Bristol, BS8 2BN UK; 5 Beth Israel Deaconess Medical Center, Boston, MA USA; 6 Department of Surgery, University Hospitals St. John Medical Center, Westlake, OH USA; 7 Department of Surgery, University of Washington, Seattle, WA USA; 8 Section for Surgical Research, Department of Surgery, Medical University of Graz, Graz, Austria; 9 Department of Surgery and Division of Gastroenterology, The Guthrie Clinic, Sayre, PA USA; 10 Department of Surgery, Mayo Clinic Florida, Jacksonville, FL USA; 11 Department of Surgery, Sidney Kimmel Medical College, Thomas Jefferson University, Philadelphia, PA USA; 12 Office of Strategic Business Development and Partnerships, Thomas Jefferson University and Jefferson Health, Philadelphia, PA USA

**Keywords:** Cholecystectomy, Bile leak, Bile duct injury, Laparoscopy, Open, Outcomes

## Abstract

**Background:**

Laparoscopic cholecystectomy (LC), one of the most commonly performed surgical procedures, remains associated with significant major morbidity including bile leak and bile duct injury (BDI). The effect of changes in practice over time, and of interventions to improve patient safety, on morbidity rates is not well understood. The aim of this review was to describe current incidence rates and trends for BDI and other complications during and after LC, and to identify risk factors and preventative measures associated with morbidity and BDI.

**Methods:**

PubMed, MEDLINE, and Web of Science database searches and data extraction were conducted for studies which reported individual complications and complication rates following laparoscopic cholecystectomy in a representative population. Outcomes data were pooled. Meta-regression analysis was performed to assess factors associated with conversion, morbidity, and BDI rates.

**Results:**

One hundred and fifty-one studies reporting outcomes for 505,292 patients were included in the final quantitative synthesis. Overall morbidity, BDI, and mortality rates were 1.6–5.3%, 0.32–0.52%, and 0.08–0.14%, respectively. Reported BDI rates reduced over time (1994–1999: 0.69(0.52–0.84)% versus 2010–2015 0.22(0.02–0.40)%, *p* = 0.011). Meta-regression analysis suggested higher conversion rates in developed versus developing countries (4.7 vs. 3.4%), though a greater degree of reporting bias was present in these studies, with no other significant associations identified.

**Conclusions:**

Overall, trends suggest a reduction in BDI over time with unchanged morbidity and mortality rates. However, data and reporting are heterogenous. Establishment of international outcomes registries should be considered.

**Electronic supplementary material:**

The online version of this article (10.1007/s00464-017-5974-2) contains supplementary material, which is available to authorized users.

Laparoscopy has become the gold standard approach to cholecystectomy since its introduction 30 years ago, and is one of the most commonly performed general surgical procedures [1]. Despite the advantages of laparoscopy, however, up to a five-fold increase in rates of bile duct injury (BDI) was reported at the onset of the era of laparoscopic cholecystectomy (LC) [2]. Relatively high rates of BDI continue to be conveyed, with individual reports ranging from 0.2 to 1.5% [3–7] suggesting that little improvement in outcomes has occurred since the introduction of LC. This compares unfavorably to the era of open surgery, where BDI rates of 0.1–0.2% were commonly accepted [2].

BDI has a substantial negative impact on patient survival, [8, 9] is associated with impaired quality of life, [10] and represents a major source of litigation cost in many modern health systems [11]. It, therefore, remains a critical goal to reduce rates of BDI, which still is the most feared complication of this common procedure conducted for benign disease.

The only previous study to analyze LC outcomes on a broad scale was an early review published in 1996 by Shea et al. [12] which pooled data for 78,747 patients from 98 studies. They found an overall collective BDI rate that ranged from 0.36 to 0.47%.

An updated comprehensive understanding of modern-day practice including morbidity and BDI rates, and any factors which may predispose or prevent complications, is necessary if practice is to be improved.

The aims of this review are twofold: (1) to describe current incidence and trends for BDI and other complications during and after LC, and (2) to identify risk factors and preventative measures associated with morbidity and BDI.

## Methods

### Search strategy

A systematic review was conducted in accordance with PRISMA guidelines for the reporting of systematic reviews and meta-analysis of observational studies [13]. PubMed, MEDLINE, and Web of Science database searches and data extraction were conducted from 1987 (first published report of laparoscopic cholecystectomy) to January 2015. The following search terms and MeSH headings were used and combined with AND operands: “cholecystectomy,” “morbidity,” “laparoscopy.” Following de-duplication, initial titles and abstracts were reviewed to identify articles of potential interest; these were then retrieved in full-text format for review and data extraction by three independent researchers. Any discrepancies during the search were discussed and revised until consensus was reached.

### Selection criteria

Studies were included only if they reported detailed individual complications and complication rates. Complications were recorded as defined by the individual study authors. An “all comers only” approach to inclusion in this review was applied, with the aim of including data representative of routine practice of LC (at time of publication) in the general population. As such, studies of select cohorts, such as analyses of LC in acalculous cholecystitis or in geriatric populations, were excluded. Studies were required to include at least 100 subjects, and those explicitly describing early case experience or learning curves were not included to ensure that data were representative of an established practice. Reports where techniques other than conventional multi-port LC (e.g., single incision; robotic) were described were included only if the technique reported represented standard practice for the reporting center or were reported within the context of a randomized trial. This approach was chosen to reduce the risk of selection bias from these studies. When two or more studies shared overlapping data sources, only one study was included according to the following prioritization criteria: (1) the most detailed relevant outcomes, (2) the largest patient population, and (3) the most recent dataset. Study, patient, procedure, and outcome variables were extracted.

### Data analysis

Descriptive and outcomes data were pooled. Ranges were calculated for pooled major outcomes, taking into account that a lack of a reported outcome did not necessarily mean its absence. For these, either only studies that explicitly reported a given outcome (higher outcome range) were used, or alternatively, all studies were included and assumed a 0% rate for those that did not report the outcome in question (lower range, i.e., “best case scenario”), replicating prior methodology [12]. Unadjusted analysis of morbidity and BDI rates over time was conducted with studies grouped into 5-year intervals, based on the last reported year of data collection.

To identify underlying factors potentially associated with variation in LC outcomes across studies, meta-regression analysis was conducted for conversion, morbidity, and BDI rates, adjusting for the following co-variates: (1) data year (as represented by the latest year of included data), (2) country of origin (developed vs. developing country according to United Nations human development index classification [14]), (3) academic *vs*. non-academic center, (4) acute cholecystectomy rate, (5) cholecystitis rate, (6) intraoperative cholangiogram rate, and (7) surgical technique (e.g., standard multi-port LC, single incision LC; each technique separately coded).

Given the inclusion of several studies assessing interventions to reduce BDI, secondary meta-analysis was performed for these papers, but was not included in the main text of this manuscript as the search strategy was not set up to capture these papers and large heterogeneity of data could be expected. Analysis was performed using Stata 12 (StataCorp, Austin, TX), using metan and metareg commands.

### Study quality assessment

Study quality was assessed using the Newcastle-Ottawa Scale [15] for cohort studies, and the Cochrane Collaboration’s Risk of Bias Assessment Tool for randomized trials [16]. Publication bias for BDI rates was assessed using funnel plots with pseudo-confidence intervals,[17] and Egger’s test.

## Results

### Search results

**Fig. 1:**
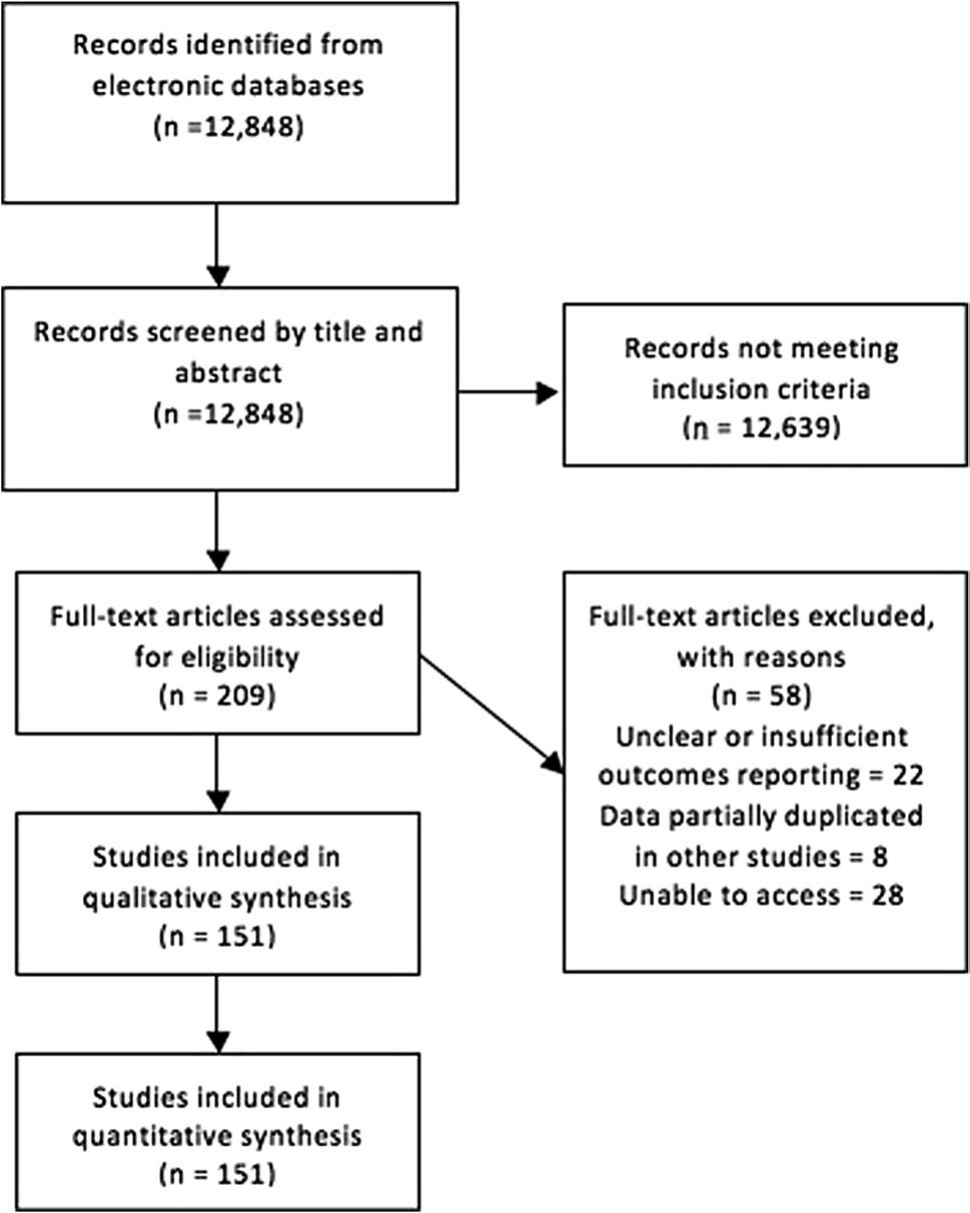
Literature search flow diagram

A total of 12,848 search results were returned and screened for eligibility (Fig. 1). Following review of title and abstracts, full-text versions of 209 articles were retrieved and reviewed in full. Of these, 151 were included in the final qualitative and quantitative analysis (see appendix 1 for full list).

A total of 505,292 patients were included in the 151 analyzed studies, which included 18 randomized trials (4549 patients), 117 single center cohort studies (115,237 patients), and 35 multi-center cohort studies (385,506 patients), with a median of 435 patients per study [interquartile range (IQR) 152–1353].

### Pooled outcomes

BDI was divided into major bile duct injuries (as defined by individual authors) and bile leaks in 65 (43%) of studies, representing 170,059 patients. For these patients, overall reported prevalence of major injury was 0.28% and bile leak 0.46%. The remaining studies reported BDI without indication of severity or anatomy. Overall, the pooled range for any biliary injury was 0.32–0.52%, with overall morbidity and mortality of 1.6–5.3 and 0.08–0.14%, respectively (Table 1). Overall pooled prevalences for individual complications are shown in Table 2.

**Table 1:** Pooled outcome data for all included studies

Outcome	Pooled range	Studies reported	Patients reported
Bile duct injury	0.32–0.52%	106 (70.1%)	307,788 (60.9%)
Morbidity	1.6–5.3%	79 (52%)	156,009 (30.9%)
Mortality	0.08–0.14%	71 (46.7%)	305,457 (60.5%)
IOC rate	5.69–26.3%	55 (36.2%)	109,202 (21.6%)
Conversion rate	4.2–6.2%	130 (85.5%)	347,803 (68.8%)

Note range of values indicates possible outcome rates if either including only studies explicitly reporting the given outcome, or assuming a 0% rate for a given outcome if not reported in pooled values

*IOC* intraoperative cholangiogram

**Table 2:** Pooled data for reported complications other than bile duct injury after laparoscopic cholecystectomy

Complication type	Prevalence (%)	Studies reported	Patients reported
Wound infection	1.25	84 (55.3%)	122,963 (24.3%)
Urinary retention	0.90	25 (16.4%)	25,863 (5.1%)
Bleeding	0.79	86 (56.6%)	146,712 (29%)
Retained CBD stones	0.50	45 (29.6%)	111,674 (22.1%)
Respiratory	0.48	40 (26.3%)	91,179 (18%)
Cardiac	0.36	32 (21.1%)	50,862 (10.1%)
Intraabdominal abscess	0.34	38 (25%)	60,517 (12%)
Hernia	0.21	33 (21.7%)	58,849 (11.6%)
Bowel injury	0.15	44 (28.9%)	99,102 (19.6%)
Sepsis	0.14	20 (13.2%)	65,123 (12.9%)
Pancreatitis	0.14	18 (11.8%)	39,453 (7.8%)
DVT/PE	0.13	14 (9.2%)	18,070 (3.6%)
Prolonged ileus	0.04	17 (11.2%)	54,150 (10.7%)

*CBD* common bile duct, *DVT*/*PE* deep vein thrombosis/pulmonary embolus

### Trends over time

**Fig. 2:**
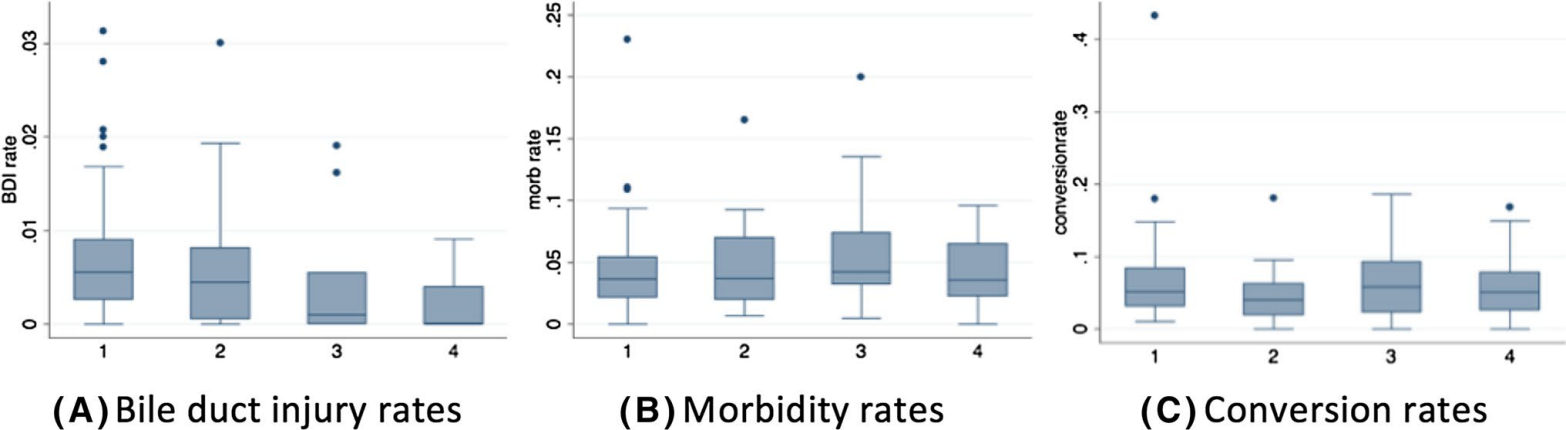
Boxplots for **A** bile duct injury rates, **B** morbidity rates, and **C** conversion rates, for each 5-year interval of included data

Unadjusted analysis demonstrated a statistically significant reduction in reported BDI from 0.69% (0.52–0.84) to 0.22% (0.02–0.40) (mean and 95% confidence interval (CI)) (*p* = 0.011) for the intervals 1994–1999 and 2010–2014, respectively. There was no statistically significant difference for conversion rates (pooled range 4.2–6.2%, *p* = 0.269), or morbidity rates (1.6–5.3%, *p* = 0.931) across time periods (Fig. 2).

### Meta-regression analysis

Multi-variate adjusted (meta-regression) analysis (Table 3) found few detectable differences in BDI or morbidity rates. For conversion rates, LC in developing countries were less likely to be converted to open (mean 3.4 vs. 4.7%, *p* = 0.026).

**Table 3:** Results of meta-regression analysis

Outcome	Variable	Coefficient	Lower 95% CI	Upper 95% CI	p value
BDI rate	Data year	− 0.009	− 0.047	0.027	0.602
	Academic center	0.165	− 0.225	0.556	0.403
	Acute cholecystectomy rate	1.318	− 5.922	8.559	0.699
	Cholecystitis rate	− 0.701	− 4.622	3.220	0.718
	IOC rate	0.061	− 0.777	0.899	0.883
	Surgical technique	0.496	− 0.308	1.299	0.224
	Country of origin	− 0.002	− 0.435	0.432	0.994
Morbidity rate	Data year	0.0166	− 0.016	0.049	0.319
	Academic center	0.256	− 0.134	0.645	0.195
	Acute cholecystectomy rate	2.231	− 1.239	5.702	0.191
	Cholecystitis rate	− 1.009	− 5.649	3.631	0.659
	IOC rate	0.249	− 0.787	1.285	0.625
	Surgical technique	− 0.453	− 1.277	0.373	0.278
	Country of origin	0.324	− 0.189	0.836	0.213
Conversion rate	Data year	− 0.017	− 0.040	0.006	0.149
	Academic center	− 0.083	− 0.354	0.187	0.545
	Acute cholecystectomy rate	1.621	− 0.665	3.906	0.152
	Cholecystitis rate	− 0.982	− 3.839	1.875	0.493
	IOC rate	0.322	− 0.366	1.010	0.351
	Surgical technique	− 0.13	− 0.776	0.516	0.692
	Country of origin^a^	0.346	0.041	0.652	0.026

*BDI* bile duct injury, *CI* confidence interval, *IOC* intraoperative cholangiogram

^a^*p* < 0.05

Considering studies that assessed the effects of interventions on LC outcomes, reporting was heterogenous and quality of studies suitable for meta-analysis was poor. No more than two studies reported comparable outcomes for any given intervention, precluding any meaningful meta-analysis or interpretation of results; these are, therefore, not further discussed in this paper, though results are included for completeness (see Appendix 2).

### Study quality and bias

Overall quality of the included studies was moderate. Newcastle-Ottawa scores for cohort studies were (mean ± standard deviation) 6.2 ± 1.0, range 4–9. Risk of bias for randomized trials using the Cochrane Collaboration tool was generally low (Appendix 1). The rate of outcome reporting was highly variable and is reported in Table 4.

**Table 4:** Rates of demographic and outcome data reporting by included studies

	Studies reported	Patients reported
Conversion rate	130 (85.5%)	347,803 (68.8%)
Gender	119 (78.3%)	207,071 (41%)
Age	111 (73%)	217,607 (43.1%)
BDI	106 (71%)	307,788 (60.9%)
Technique	84 (55.3%)	93,035 (18.4%)
Morbidity	79 (52%)	156,009 (30.9%)
Duration of hospital stay	75 (49.3%)	224,955 (44.5%)
Mortality	71 (46.7%)	305,457 (60.5%)
Operative time	69 (45.4%)	79,452 (15.7%)
IOC rate	55 (36.2%)	109,202 (21.6%)
Cholecystitis rate	35 (23%)	51,749 (10.2%)
BMI	23 (15.1%)	27,567 (5.5%)
Acute cholecystectomy	18 (11.8%)	42,866 (8.5%)

*BDI* bile duct injury, *IOC* intraoperative cholangiogram, *BMI* body mass index

**Fig. 3:**
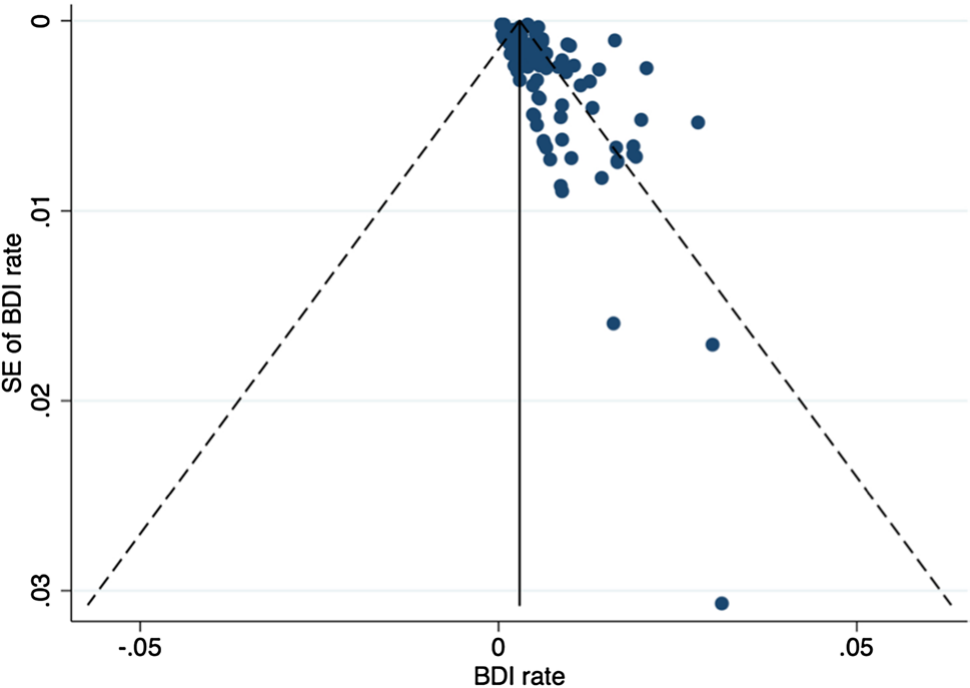
Funnel plot for bile duct injury rates

**Fig. 4:**
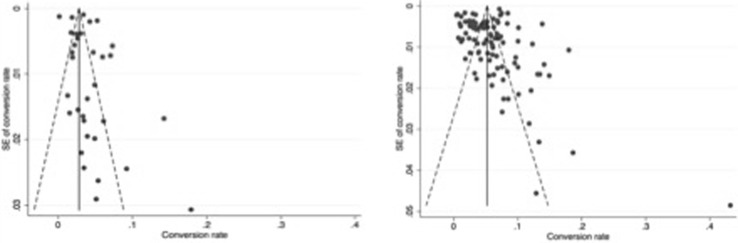
Comparison of funnel plots for conversion rates for developing (left) and developed (right) countries

Publication bias for BDI rates using funnel plots and pseudo-confidence intervals are found in Figs. 3 and 4. Use of Egger’s test suggested statistically significant bias (bias coefficient 1.87 ± 0.34, *p* < 0.001). Bias risk for reported conversion rates was also assessed for developed (0.13 ± 1.02, *p* = 0.898) and developing countries (1.42 ± 1.02, *p* = 0.178) of origin.

## Discussion

This review of outcomes for LC reconfirms the established principle that LC is safe and may be performed with minimum morbidity (pooled prevalence range 1.6–5.3%) and mortality (0.08–0.14%). LC remains the unchallenged gold standard, with conversion rates between 4.2 and 6.2%.

BDI outcomes analysis was limited by the fact that BDI was not universally reported and different classifications were used (without any formalized correspondence as to the grading); we were, therefore, unable to segregate bile leaks according to severity or nature of injury. Our findings, however, are consistent with recent Swedish population analyses of over 51,000 LCs which reported a 0.3% major BDI rate, [18] with an overall 1.5% bile leak rate [7]. At 0.32%, the lower estimate of pooled BDI rate in our overall analysis remained higher than the commonly accepted rates of 0.1–0.2% for open cholecystectomy reported at the dawn of the laparoscopic era over a quarter century ago [2]. Unadjusted pooled outcomes in our study suggested a modest decrease in reported BDI rates over the past 30 years. However, this was not reflected in the meta-regression analysis. Regardless, with nearly 1 million cholecystectomies performed per year in the United States alone, [19] the medical, psychological, and socioeconomic burdens represented by BDI remain substantial. This consideration is particularly true if one weighs it in the context of LC as an extremely common, and, by today’s standards, a typically outpatient or short stay procedure performed for benign disease. Furthermore, a large volume of performed LC are probably not included in any studies and their complication rate may be higher than the ones presented here. Yamashita et al., for example, reported Japanese national survey data suggesting little change in BDI rates over the past decade, with a mean incidence of 0.66% [20].

Technological and technical efforts to improve LC quality continue. However, despite the growing number of methods intended to reduce BDI, the evidence for their ability to impact BDI rates remains limited. A 2011 systematic review of interventions to prevent BDI identified a number of candidate techniques and procedures, including routine use of the critical view of safety approach and intraoperative cholangiography, but conclusive effects on BDI rates could not be shown [21]. In addition, series which have examined the mechanism of biliary injury have rarely described use of the critical view of safety as the method of ductal identification [22, 23]. However, several large retrospective series in which the critical view of safety was used routinely have been reported with no biliary injuries [24, 25].

A major challenge for assessing factors associated with BDI risk is its relatively low prevalence (0.3–0.5%). A study assessing a procedure to reduce BDI rates would, for example, require a sample size of 16,989 patients in each arm to detect a 50% reduction of the BDI rates from 0.3% (assuming a standard alpha = 0.05 at 80% power). The logistical obstacles and confounders involved in a study of this size means such a trial is highly unlikely to take place. Moreover, the true prevalence of BDI is difficult to ascertain from the literature, as there is wide confusion between prevalence and incidences in reports. Instead, we must, therefore, rely on cohort, expert, and surrogate data, such as the ability to accurately demonstrate and identify biliary anatomy intraoperatively, as is the aim with the critical view of safety, and other methods of ductal identification [26, 27]. Newer techniques such as infrared fluorescence cholangiography are generating considerable interest as a way to enhance identification of the anatomy during cholecystectomy, but must be evaluated by further study in larger numbers of patients before any recommendations can be made [28].

In the present study, we assessed the effect of technical, patient, and hospital characteristics on BDI rates. These factors had few detectable associations with BDI rates. It is crucial, therefore, to recognize the role that education, decision-making, and experience have to play in preventing BDI during LC. A recent insurance database review study of BDI illustrated the effect of surgeon experience and volume, wherein younger, less experienced surgeons reported BDI rates three times higher than their more experienced counterparts [29]. A European-based population study has suggested that a positive volume–outcome relationship for LC exists with reference to outcomes other than BDI as well, with high volume centers (defined as > 244 LC/year) reporting lower morbidity, mortality, and reoperation rates [30].

Data published by Way et al., [31] in which root causes of over 200 BDIs were identified, highlighted the fact that over 97% of errors were related to non-technical, predominantly perceptual errors. In response to this and other data, a recent international Delphi consensus study headed by the Society for American Gastrointestinal Endoscopic Surgeons (SAGES) Safe Cholecystectomy Task Force identified the most important factors to safety in LC as (1) establishing the CVS, (2) understanding of anatomy, (3) adequate exposure, (4) ability to call a senior colleague for help, and (5) recognizing when to convert or abandon [32]. Education and practice, the group recommended, should be focused around these key principles, with technical factors such as choice of dissection tool, duct securing technique, or use of cholangiography ranking lower in priority.

This review and its conclusions are subject to several limitations. As discussed, we had limited statistical power to identify differences in BDI rates by a range of factors because of its relatively low prevalence and/or incidence rates. Secondly, we imposed minimum sample sizes to reduce the risk of selection bias from smaller studies. This approach may have had the effect of excluding studies that might otherwise have been included in secondary outcome analyses, and meant that no more than two studies were available for analysis of each assessed intervention. It was, however, our expressed aim to focus on BDI and complication rates and numerous other reviews (including those already cited) for individual interventions and secondary outcomes exist which incorporated smaller studies not included here. The ranges reported here for pooled outcomes reflect the inconsistency of data reporting, even for large-scale studies focused on outcomes for LC. For the lower range, assuming a 0% prevalence or incidence for studies which did not report a given outcome will invariably underestimate the pooled rate as it combines studies which did not report prevalence/incidence due to a true 0% rate, with those with incomplete outcomes reporting. Interpretation of our findings must take this aspect into account.

In addition, it is almost certain that many BDI are neither reported nor published except for national database reporting requirements such as in Sweden, and therefore, the true prevalence/incidence of BDI in most of the world is not precisely known and may be higher than identified herein. Administrative databases accounted for a large proportion of the retrospective studies included here; their advantageous large numbers are tempered by the fact that they likely lack detailed coding with reference to prevalence/incidence of BDI, and do not allow differentiation between types of injury. Further, if BDIs are recorded without severity, based on reoperation (hepaticojejunostomy) alone, this surrogate endpoint presents the dual problem of skewing injuries to the severe end of the scale, as well as underestimating overall injury rates—potentially accounting in part for the apparent reduction observed in our pooling of recent data of BDI.

Although our inclusion criteria aimed to maintain high overall study quality, the thoroughness of outcomes reporting was highly variable. In many cases, the quality of reporting in recent literature was worse than 20 years ago; duration of stay and morbidity were reported for 49 and 52% of studies included, respectively, compared to 82 and 98% in the previous 1996 analysis by Shea et al. [12]. This finding highlights the need for standardized reporting of outcomes for these procedures [33]. Finally, analysis of funnel plots suggested significant publication bias. This finding potentially also accounts for the differences seen in conversion rates between developed and developing countries, with a much higher bias coefficient seen in developing countries.

Future advances to improve outcomes in LC will need to consider technological, educational, and structural approaches. Technological developments offer new techniques for the identification of biliary anatomy but have limited evidence demonstrating their effectiveness. Though some show promise, it is unlikely that interventions will be able to detect an effect on BDI within the context of a randomized trial due to limitations of sample size. It is incumbent upon researchers, therefore, to identify and focus on selected surrogate outcome measures, and particularly to ensure the standardization of reporting, for example including complications, BDI, and ductal injury type, which continues to elude LC-related research. The establishment of international registries should be prioritized.

Education, training, and mentorship, with emphasis on techniques such as the critical view of safety, will continue to be the mainstay of surgical expertise. Modern teaching paradigms have enhanced educators’ understanding of the often unconsciously carried knowledge which constitutes surgical expertise, with teaching frameworks designed to reveal and convey these to learners [34]. Aided by technological teaching adjuncts, including virtual reality and box trainers, these have the potential to accelerate improvement and abbreviate learning curves [35]. The technology, training, and expertise required for LC, coupled with the severity of BDI as a potential complication, calls for a reassessment of how LC is taught and what educational strategies could be used to impact this problem.

## Conclusions

This extensive systematic review and pooled data analysis summarizes the current body of knowledge relating to outcomes following laparoscopic cholecystectomy. It could represent a useful benchmark against which clinicians and health systems may measure outcomes, with which patients may weigh the risks of surgery, and against which researchers may assess their data reporting.

No definitive intervention to reduce BDI rates was identified, which likely reflects the limitations of the data reported in these various studies. Overall, we report marginal, if any, reductions in the rate of reported BDI. Pooled rates for BDI after LC remain higher than during the era of open cholecystectomy. Given the high prevalence of cholecystectomy, thousands of patients per year continue to sustain BDI, with severe and long-term implications for their health, underscoring once again the need to continue research in this field, and to inform and educate young surgeons concerning the risk and consequences of BDI during LC.

## Electronic supplementary material

Below is the link to the electronic supplementary material.

Supplementary material 1 (DOCX 171 KB)

Supplementary material 2 (DOCX 8089 KB)

## References

[1] Shaffer EA (2006). Gallstone disease: Epidemiology of gallbladder stone disease. Best Pract Res Clin Gastroenterol.

[2] Club SS (1991). A prospective analysis of 1518 laparoscopic cholecystectomies. The Southern Surgeons Club. N Engl J Med.

[3] Harboe KM, Bardram L (2011). The quality of cholecystectomy in Denmark: outcome and risk factors for 20,307 patients from the national database. Surg Endosc.

[4] Waage A, Nilsson M (2006). Iatrogenic bile duct injury: a population-based study of 152 776 cholecystectomies in the Swedish inpatient registry. Arch Surg.

[5] Flum DR, Dellinger EP, Cheadle A, Chan L, Koepsell T (2003). Intraoperative cholangiography and risk of common bile duct injury during cholecystectomy. JAMA.

[6] Navez B, Ungureanu F, Michiels M, Claeys D, Muysoms F, Hubert C, Vanderveken M, Detry O, Detroz B, Closset J, Devos B, Kint M, Navez J, Zech F, Gigot JF, Belgian Group for Endoscopic S, the H, Pancreatic Section of the Royal Belgian Society of S (2012). Surgical management of acute cholecystitis: results of a 2-year prospective multicenter survey in Belgium. Surg Endosc.

[7] Tornqvist B, Stromberg C, Akre O, Enochsson L, Nilsson M (2015). Selective intraoperative cholangiography and risk of bile duct injury during cholecystectomy. Br J Surg.

[8] Tornqvist B, Zheng Z, Ye W, Waage A, Nilsson M (2009). Long-term effects of iatrogenic bile duct injury during cholecystectomy. Clin Gastroenterol Hepatol.

[9] Pucher PH, Aggarwal R, Qurashi M, Darzi A (2014). Meta-analysis of the effect of postoperative in-hospital morbidity on long-term patient survival. Br J Surg.

[10] Bouras G, Burns EM, Howell AM, Bagnall NM, Lee H, Athanasiou T, Darzi A (2014). Systematic review of the impact of surgical harm on quality of life after general and gastrointestinal surgery. Ann Surg.

[11] Berci G, Hunter J, Morgenstern L, Arregui M, Brunt M, Carroll B, Edye M, Fermelia D, Ferzli G, Greene F, Petelin J, Phillips E, Ponsky J, Sax H, Schwaitzberg S, Soper N, Swanstrom L, Traverso W (2013). Laparoscopic cholecystectomy: first, do no harm; second, take care of bile duct stones. Surg Endosc.

[12] Shea JA, Healey MJ, Berlin JA, Clarke JR, Malet PF, Staroscik RN, Schwartz JS, Williams SV (1996). Mortality and complications associated with laparoscopic cholecystectomy. a meta-analysis. Ann Surg.

[13] Liberati A, Altman DG, Tetzlaff J, Mulrow C, Gotzsche PC, Ioannidis JP, Clarke M, Devereaux PJ, Kleijnen J, Moher D (2009). The PRISMA statement for reporting systematic reviews and meta-analyses of studies that evaluate healthcare interventions: explanation and elaboration. BMJ.

[14] United Nations Development Programme Human Development Reports (2010) International human development indicators. http://hdr.undp.org/en/countries. Accessed 1 Sept 2016

[15] Wells GA, Shea B, O’Connell B, Peterson J, Welch V, Losos M, Tugwell P (2014) The Newcastle-Ottawa Scale (NOS) for assessing the quality of nonrandomised studies in meta-analyses. http://www.ohri.ca/programs/clinical_epidemiology/oxford.asp. Accessed 1 Jul 2014

[16] Cochrane Collaboration (2014) The Cochrane Collaboration’s tool for assessing risk of bias. http://ohg.cochrane.org/sites/ohg.cochrane.org/files/uploads/Risk of bias assessment tool.pdf. Accessed 1 Jul 2014

[17] Agresti A, Ryu E (2010). Pseudo-score confidence intervals for parameters in discrete statistical models. Biometrika.

[18] Rystedt J, Lindell G, Montgomery A (2016). Bile duct injuries associated with 55,134 cholecystectomies: treatment and outcome from a national perspective. World J Surg.

[19] US Department of Health and Human Services, National Center for Health Statistics (2009). National Health Statistics Reports: Ambulatory Surgery in the United States.

[20] Yamashita Y, Takada T, Strasberg SM, Pitt HA, Gouma DJ, Garden OJ, Buchler MW, Gomi H, Dervenis C, Windsor JA, Kim SW, De Santibanes E, Padbury R, Chen XP, Chan ACW, Fan ST, Jagannath P, Mayumi T, Yoshida M, Miura F, Tsuyuguchi T, Itoi T, Supe AN (2013). TG13 surgical management of acute cholecystitis. J Hepatol Biliary Pancreat Sci.

[21] Buddingh KT, Nieuwenhuijs VB, van Buuren L, Hulscher JB, de Jong JS, van Dam GM (2011). Intraoperative assessment of biliary anatomy for prevention of bile duct injury: a review of current and future patient safety interventions. Surg Endosc.

[22] Nijssen MA, Schreinemakers JM, Meyer Z, van der Schelling GP, Crolla RM, Rijken AM (2015). Complications after laparoscopic cholecystectomy: a video evaluation study of whether the critical view of safety was reached. World J Surg.

[23] Booij KA, de Reuver PR, Nijsse B, Busch OR, van Gulik TM, Gouma DJ (2014). Insufficient safety measures reported in operation notes of complicated laparoscopic cholecystectomies. Surgery.

[24] Yegiyants S, Collins JC (2008). Operative strategy can reduce the incidence of major bile duct injury in laparoscopic cholecystectomy. Am Surg.

[25] Avgerinos C, Kelgiorgi D, Touloumis Z, Baltatzi L, Dervenis C (2009). One thousand laparoscopic cholecystectomies in a single surgical unit using the “critical view of safety” technique. J Gastrointest Surg.

[26] Strasberg SM, Hertl M, Soper NJ (1995). An analysis of the problem of biliary injury during laparoscopic cholecystectomy. J Am Coll Surg.

[27] Strasberg SM, Brunt LM (2017). The critical view of safety: why it is not the only method of ductal identification within the standard of care in laparoscopic cholecystectomy. Ann Surg.

[28] Osayi SN, Wendling MR, Drosdeck JM, Chaudhry UI, Perry KA, Noria SF, Mikami DJ, Needleman BJ, Muscarella P, Abdel-Rasoul M, Renton DB, Melvin WS, Hazey JW, Narula VK (2015). Near-infrared fluorescent cholangiography facilitates identification of biliary anatomy during laparoscopic cholecystectomy. Surg Endosc.

[29] Schwaitzberg SD, Scott DJ, Jones DB, McKinley SK, Castrillion J, Hunter TD, Michael Brunt L (2014). Threefold increased bile duct injury rate is associated with less surgeon experience in an insurance claims database: more rigorous training in biliary surgery may be needed. Surg Endosc.

[30] Harrison EM, O’Neill S, Meurs TS, Wong PL, Duxbury M, Paterson-Brown S, Wigmore SJ, Garden OJ (2012). Hospital volume and patient outcomes after cholecystectomy in Scotland: retrospective, national population based study. BMJ.

[31] Way LW, Stewart L, Gantert W, Liu K, Lee CM, Whang K, Hunter JG (2003). Causes and prevention of laparoscopic bile duct injuries: analysis of 252 cases from a human factors and cognitive psychology perspective. Ann Surg.

[32] Pucher PH, Brunt LM, Fanelli RD, Asbun HJ, Aggarwal R (2015). SAGES expert Delphi consensus: critical factors for safe surgical practice in laparoscopic cholecystectomy. Surg Endosc.

[33] Cho JY, Jaeger AR, Sanford DE, Fields RC, Strasberg SM (2015). Proposal for standardized tabular reporting of observational surgical studies illustrated in a study on primary repair of bile duct injuries. J Am Coll Surg.

[34] McKinley SK, Brunt LM, Schwaitzberg SD (2014). Prevention of bile duct injury: the case for incorporating educational theories of expertise. Surg Endosc.

[35] Aggarwal R, Crochet P, Dias A, Misra A, Ziprin P, Darzi A (2009). Development of a virtual reality training curriculum for laparoscopic cholecystectomy. Br J Surg.

