# Supplementary material for: Outcome trends and safety measures after 30 years of laparoscopic cholecystectomy: a systematic review and pooled data analysis
Source: Surg Endosc. 2018 Mar 19;32(5):2175–83. doi: 10.1007/s00464-017-5974-2 (PMC5897463; doi:10.1007/s00464-017-5974-2)
Supplement: Supplementary file 1 — Supplementary material 1 (DOCX 171 KB) [file 464_2017_5974_MOESM1_ESM.docx]

**Appendix** **1.** List of included references, including study quality assessment utilising Newcastle Ottawa Score (NOS) for non-randomised studies, and Cochrane Risk of Bias Tool categories of bias (selection, performance, detection, reporting, and attrition) for randomised trials, and reporting number of included patients, n. Note numeric label in this table does not equate to reference number in manuscript text.

|  | **1st Author** | **Year** | **Title** | **Journal** | **Vol** | **No** | **Pages** | **NOS** | **Selection - randomisation** | **Selection - allocation concealment** | **Performance - blinded participants** | **Detection - blinded outcome assessment** | **Reporting - selective reporting** | **Attrition - follow up** | **n** |
| --- | --- | --- | --- | --- | --- | --- | --- | --- | --- | --- | --- | --- | --- | --- | --- |
| 1 | Raman SR | 2012 | The degree of gallbladder wall thickness and its impact on outcomes after laparoscopic cholecystectomy | Surgical Endoscopy and Other Interventional Techniques | 26 | 11 | 3174-3179 | 6 |  |  |  |  |  |  | 874 |
| 2 | Donkervoort SC | 2012 | Outcome of laparoscopic cholecystectomy conversion: Is the surgeon's selection needed? | Surgical Endoscopy and Other Interventional Techniques | 26 | 8 | 2360-2366 | 7 |  |  |  |  |  |  | 1126 |
| 3 | Suo G | 2013 | Clipless minilaparoscopic cholecystectomy: a study of 1,096 cases | Journal of Laparoendoscopic & Advanced Surgical Techniques Part A | 23 | 10 | 849-854 | 4 |  |  |  |  |  |  | 1096 |
| 4 | Wills E | 2013 | Clipless versus conventional laparoscopic cholecystectomy | Journal of Laparoendoscopic & Advanced Surgical Techniques Part A | 23 | 3 | 237-239 | 5 |  |  |  |  |  |  | 208 |
| 5 | Neureuther SJ | 2013 | The effect of insurance status on outcomes after laparoscopic cholecystectomy | Surgical Endoscopy | 27 | 5 | 1761-1765 | 7 |  |  |  |  |  |  | 1090 |
| 6 | Marks JM | 2013 | Single-incision laparoscopic cholecystectomy is associated with improved cosmesis scoring at the cost of significantly higher hernia rates: 1-year results of a prospective randomized, multicenter, single-blinded trial of traditional multiport laparoscopic cholecystectomy vs single-incision laparoscopic cholecystectomy | Journal of the American College of Surgeons | 216 | 6 | 1037-1047 |  | low | low | low | low | low | low | 200 |
| 7 | Rosenmuller MH | 2013 | Expertise-based randomized clinical trial of laparoscopic versus small-incision open cholecystectomy | British Journal of Surgery | 100 | 7 | 886-894 |  | low | low | high | high | low | low | 333 |
| 8 | Kamran K | 2013 | Does sex affect the outcome of laparoscopic cholecystectomy? A retrospective analysis of single center experience | Asian Journal of Endoscopic Surgery | 6 | 1 | 21-25 | 6 |  |  |  |  |  |  | 2061 |
| 9 | Andrews S | 2013 | Does concentration of surgical expertise improve outcomes for laparoscopic cholecystectomy? 9 year audit cycle | The Surgeon, Journal of the Royal Colleges of Surgeons of Edinburgh & Ireland | 11 | 6 | 309-312 | 7 |  |  |  |  |  |  | 1605 |
| 10 | Tiwari S | 2013 | Laparoscopic cholecystectomy under spinal anaesthesia: A prospective, randomised study | Journal of Minimal Access Surgery | 9 | 2 | 65-71 |  | low | high | high | low | low | low | 114 |
| 11 | Tian MG | 2013 | Two-port laparoscopic cholecystectomy with modified suture retraction of the fundus: A practical approach | Journal of Minimal Access Surgery | 9 | 3 | 122-125 |  |  |  |  |  |  |  | 107 |
| 12 | Farkas DT | 2012 | The impact of body mass index on outcomes after laparoscopic cholecystectomy | Surgical Endoscopy | 26 | 4 | 964-969 | 6 |  |  |  |  |  |  | 1027 |
| 13 | Sasaki K | 2012 | Original single-incision laparoscopic cholecystectomy for acute inflammation of the gallbladder | World Journal of Gastroenterology | 18 | 9 | 944-951 | 5 |  |  |  |  |  |  | 110 |
| 14 | Hasbahceci M | 2012 | Laparoscopic cholecystectomy in a single, non-teaching hospital: an analysis of 1557 patients | Journal of Laparoendoscopic & Advanced Surgical Techniques Part A | 22 | 6 | 527-532 |  |  |  |  |  |  |  | 1557 |
| 15 | Comitalo JB | 2012 | Laparoscopic cholecystectomy and newer techniques of gallbladder removal | Journal of the Society of Laparoendoscopic Surgeons | 16 | 3 | 406-12 |  |  |  |  |  |  |  | 1000 |
| 16 | Ammori MB | 2012 | Laparoscopic cholecystectomy without intraoperative cholangiography | Journal of Laparoendoscopic and Advanced Surgical Techniques | 22 | 2 | 146-151 |  |  |  |  |  |  |  | 549 |
| 17 | Choi SH | 2012 | Single-fulcrum laparoscopic cholecystectomy: A single-incision and multi-port technique | ANZ Journal of Surgery | 82 | (7-8) | 529-534 | 5 |  |  |  |  |  |  | 130 |
| 18 | Vilallonga R | 2012 | Single-port transumbilical laparoscopic cholecystectomy: A prospective randomised comparison of clinical results of 140 cases | Journal of Minimal Access Surgery | 8 | 3 | 74-78 |  | low | unsure | high | high | unsure | unsure | 140 |
| 19 | Kanakala V | 2011 | Risk factors in laparoscopic cholecystectomy: a multivariate analysis | International Journal Of Surgery | 9 | 4 | 318-323 |  |  |  |  |  |  |  | 2117 |
| 20 | Khambaty F | 2011 | Laparoscopic versus single-incision cholecystectomy | World Journal of Surgery | 35 | 5 | 967-972 | 8 |  |  |  |  |  |  | 107 |
| 21 | Harboe KM | 2011 | The quality of cholecystectomy in Denmark: outcome and risk factors for 20,307 patients from the national database | Surgical Endoscopy | 25 | 5 | 1630-1641 | 8 |  |  |  |  |  |  | 20307 |
| 22 | Buddingh KT | 2011 | Lower rate of major bile duct injury and increased intraoperative management of common bile duct stones after implementation of routine intraoperative cholangiography | Journal of the American College of Surgeons | 213 | 2 | 267-274 | 7 |  |  |  |  |  |  | 421 |
| 23 | Al-Mulhim AS | 2011 | Outcome of laparoscopic cholecystectomy at a secondary level of care in Saudi Arabia | Saudi Journal of Gastroenterology | 17 | 1 | 47-52 |  |  |  |  |  |  |  | 968 |
| 24 | Jain SK | 2011 | A prospective, randomized study of comparison of clipless cholecystectomy with conventional laparoscopic cholecystectomy | Journal of Laparoendoscopic and Advanced Surgical Techniques | 21 | 3 | 203-208 |  | low | low | unsure | unsure | unsure | unsure | 100 |
| 25 | Young AL | 2010 | Index admission laparoscopic cholecystectomy for patients with acute biliary symptoms: results from a specialist centre | HPB | 12 | 4 | 270-276 | 6 |  |  |  |  |  |  | 439 |
| 26 | Cengiz Y | 2010 | Improved outcome after laparoscopic cholecystectomy with ultrasonic dissection: a randomized multicenter trial | Surgical Endoscopy | 24 | 3 | 624-630 |  | low | low | low | low | low | low | 243 |
| 27 | Thesbjerg SE | 2010 | Sex differences in laparoscopic cholecystectomy | Surgical Endoscopy | 24 | 12 | 3068-3072 | 7 |  |  |  |  |  |  | 5951 |
| 28 | Wichmann MW | 2010 | Laparoscopic cholecystectomy--comparison of early postoperative results in an Australian rural centre and a German university hospital | Langenbecks Archives of Surgery | 395 | 3 | 255-260 | 5 |  |  |  |  |  |  | 140 |
| 29 | Ghnnam W | 2010 | Rate of conversion and complications of laparoscopic cholecystectomy in a tertiary care center in Saudi Arabia | Annals of Saudi Medicine | 30 | 2 | 145-148 |  |  |  |  |  |  |  | 340 |
| 30 | Agarwal BB | 2010 | Results of laparoscopic cholecystectomy without energized dissection: a prospective study | International Journal Of Surgery | 8 | 2 | 167-172 |  |  |  |  |  |  |  | 135 |
| 31 | Ching SS | 2009 | Randomized clinical trial of torsional versus linear mode ultrasonically activated devices for laparoscopic cholecystectomy | Surgical Endoscopy | 23 | 7 | 1506-1511 | 6 |  |  |  |  |  |  | 75 |
| 32 | Priego P | 2009 | Results of laparoscopic cholecystectomy in a third-level university hospital after 17 years of experience | Revista Espanola de Enfermedades Digestivas | 101 | 1 | 20-30 | 7 |  |  |  |  |  |  | 3933 |
| 33 | Machi J | 2009 | The routine use of laparoscopic ultrasound decreases bile duct injury: a multicenter study | Surgical Endoscopy | 23 | 2 | 384-388 |  |  |  |  |  |  |  | 1381 |
| 34 | Ou ZB | 2009 | Prevention of common bile duct injury during laparoscopic cholecystectomy | Hepatobiliary & Pancreatic Diseases International | 8 | 4 | 414-417 |  |  |  |  |  |  |  | 10000 |
| 35 | Tzovaras G | 2009 | Is there a role for drain use in elective laparoscopic cholecystectomy? A controlled randomized trial | American Journal of Surgery | 197 | 6 | 759-763 |  | low | high | high | high | unsure | unsure | 284 |
| 36 | Avgerinos C | 2009 | One thousand laparoscopic cholecystectomies in a single surgical unit using the "critical view of safety" technique | Journal of Gastrointestinal Surgery | 13 | 3 | 498-503 |  |  |  |  |  |  |  | 1046 |
| 37 | Keus F | 2008 | Randomized clinical trial of small-incision and laparoscopic cholecystectomy in patients with symptomatic cholecystolithiasis: primary and clinical outcomes | Archives of Surgery | 143 | 4 | 371-378 |  | low | low | high | high | low | low | 105 |
| 38 | Veen EJ | 2008 | Outcome measurement in laparoscopic cholecystectomy by using a prospective complication registry: results of an audit | International Journal for Quality in Health Care | 20 | 2 | 144-151 |  |  |  |  |  |  |  | 1254 |
| 39 | Georgiades CP | 2008 | Is inflammation a significant predictor of bile duct injury during laparoscopic cholecystectomy? | Surgical Endoscopy | 22 | 9 | 1959-1964 |  |  |  |  |  |  |  | 2184 |
| 40 | T. Samdani, T. Singhal, A. Hussain, S. Grandy-Smith, J. Nicholls and S. El-Hasani | 2008 | Patient experience with gallstone disease in a national health service district hospital | Journal of the Society of Laparoendoscopic Surgeons | 12 | 4 | 389-94 | 6 |  |  |  |  |  |  | 1322 |
| 41 | A. W. C. Kow, A. Tan, S. P. Chang, S. F. Lee, C. P. Chan, K. H. Liau, and C. K. Ho | 2008 | An audit of ambulatory laparoscopic cholecystectomy in a Singapore institution: Are we ready for day-case laparoscopic cholecystectomy? | Hpb | 10 | 6 | 433-438 | 6 |  |  |  |  |  |  | 405 |
| 42 | I. M. Paquette, D. Smink, and S.R.G. Finlayson | 2008 | Outpatient Cholecystectomy at Hospitals Versus Freestanding Ambulatory Surgical Centers | Journal of the American College of Surgeons | 206 | 2 | 301-305 | 6 |  |  |  |  |  |  | 1496 |
| 43 | M. Tuveri and A. Tuveri | 2007 | Laparoscopic cholecystectomy: complications and conversions with the 3-trocar technique: a 10-year review | Surgical Laparoscopy, Endoscopy & Percutaneous Techniques | 17 | 5 | 380-4 | 6 |  |  |  |  |  |  | 1774 |
| 44 | C. Simoens, D. Smets, C. Ngongang and P. Mendes da Costa | 2007 | Laparoscopic cholecystectomy; a retrospective 10-year study | Hepato-Gastroenterology | 54 | 77 | 1326-30 | 6 |  |  |  |  |  |  | 1255 |
| 45 | G. Pradhan, K. Bhoomi, A. Dhital and C. L. Bhattachan | 2007 | Review of laparoscopic cholecystectomy in Nepal Medical College Teaching Hospital | Nepal Medical College Journal: NMCJ | 9 | 1 | 32-5 | 6 |  |  |  |  |  |  | 140 |
| 46 | M. Mohsin, O. Kirmani, T. Majid, K. Wani, M.-U. Hassan, J. Naqshbandi and M. Maqbool | 2007 | Is intra-operative cholangiography necessary during laparoscopic cholecystectomy? A multicentre rural experience from a developing world country | World Journal of Gastroenterology | 13 | 33 | 4493-7 | 6 |  |  |  |  |  |  | 1267 |
| 47 | P. Innocenti, G. Duzel, G. Liddo, E. Ballone and V. J. Simunovic | 2007 | Ten years of laparoscopic cholecystectomy: a comparison between a developed and a less developed country | Wiener Klinische Wochenschrift | 119 | 23-24 | 722-8 | 6 |  |  |  |  |  |  | 952 |
| 48 | S. Angelopoulos, D. Kanellos, M.-G. Pramateftakis, N. Sapidis, H. Stamatopoulos, I. Kanellos, K. Tsalis and D. Betsis | 2007 | Laparoscopic cholecystectomy without intraoperative cholangiography | Journal of Laparoendoscopic & Advanced Surgical Techniques Part A | 17 | 5 | 620-5 | 9 |  |  |  |  |  |  | 1851 |
| 49 | S. Topaloglu, S. Topcu, S. Turkyilmaz, U. Kucuktulu and B. Piskin | 2007 | Routine intraoperative aspiration of gallbladder during laparoscopic cholecystectomy | Surgical Endoscopy | 21 | 9 | 1578-81 | 8 |  |  |  |  |  |  | 100 |
| 50 | V. Neri | 2007 | Antegrade dissection in laparoscopic cholecystectomy | JSLS : Journal of the Society of Laparoendoscopic Surgeons / Society of Laparoendoscopic Surgeons | 11 | 2 | 225-228 | 6 |  |  |  |  |  |  | 127 |
| 51 | J.-M. Michel, I. Opitz, D. Th Inderbitzin, T. Kocher, L. Krahenbuhl, L. Swiss Association of and G. Thoracoscopic Surgery Study | 2006 | Risk factors for perioperative complications in patients undergoing laparoscopic cholecystectomy: analysis of 22,953 consecutive cases from the Swiss Association of Laparoscopic and Thoracoscopic Surgery database | Journal of the American College of Surgeons | 203 | 5 | 723-8 | 7 |  |  |  |  |  |  | 22953 |
| 52 | P. Whitwam, D. Turner, A. Chadha and J. Degani | 2006 | Laparoscopic cholecystectomy and management of biliary tract stones in a freestanding ambulatory surgery center | Journal of the Society of Laparoendoscopic Surgeons | 10 | 1 | 47-51 | 6 |  |  |  |  |  |  | 338 |
| 53 | S. N. Karamanakos, C. Spyropoulos, S. Panagiotopoulos, M. Karanikolas and M. Stavropoulos | 2006 | Laparoscopic cholecystectomy: a report from a single center | World Journal of Gastroenterology | 12 | 24 | 3887-90 | 6 |  |  |  |  |  |  | 1220 |
| 54 | T. Nakagawa, M. Nishioka, S. Ogata, T. Miyauchi, Y. Kashiwagi, N. Uemura and S. Inoue | 2006 | Costs and benefits of laparoscopic cholecystectomy: abdominal wall lifting vs. pneumoperitoneum procedure | Hepato-Gastroenterology | 53 | 70 | 497-500 | 6 |  |  |  |  |  |  | 207 |
| 55 | C. A. L. Thomas, T. McCartney, P. Bhoorasingh, G. Smith, P. Lodenquai and D. I. G. Mitchell | 2005 | Selective operative cholangiography in the performance of laparoscopic cholecystectomy | International Journal of Clinical Practice | 59 | 11 | 1301-3 | 5 |  |  |  |  |  |  | 194 |
| 56 | F. Giuliante, I. Giovannini, F. Ardito, F. D'Acapito, M. Vellone, M. Murazio and G. Capelli | 2005 | Bile duct injury during laparoscopic cholecystectomy: results of an Italian national survey on 56 591 cholecystectomies | Archives of Surgery | 140 | 10 | 986-92 | 7 |  |  |  |  |  |  | 56591 |
| 57 | J. Schiff, G. Rendon, J. Rothschild and S. Schwaitzberg | 2005 | Laparoscopic cholecystectomy after the learning curve: what should we expect? | Surgical Endoscopy | 19 | 9 | 1266-71 | 8 |  |  |  |  |  |  | 954 |
| 58 | U. Lepner, V. Grünthal | 2005 | Intraoperative cholangiography can be safely omitted during laparoscopic cholecystectomy: A prospective study of 413 consecutive patients | Scandinavian Journal of Surgery | 94 | 3 | 197-200 | 8 |  |  |  |  |  |  | 413 |
| 59 | J. Jarhült | 2005 | Is preoperative evaluation of the biliary tree necessary in uncomplicated gallstone disease? Results of a randomized trial | Scandinavian Journal of Surgery | 94 | 1 | 31-33 |  | Unsure | Unsure | Unsure | Unsure | Low | Unsure | 153 |
| 60 | T.M. Fullum, S. King, D. Dan, P.L. Turner | 2005 | Laparoscopic "Dome-down" cholecystectomy with the LCS-5 Harmonic scalpel | JSLS : Journal of the Society of Laparoendoscopic Surgeons / Society of Laparoendoscopic Surgeons | 9 | 1 | 51-57 | 8 |  |  |  |  |  |  | 105 |
| 61 | I.MA. Salam, A. Own, N.A. Kareem, O. A. Hameed, C. J. Yak, K. A. Zaki | 2005 | Laparoscopic cholecystectomy in the Academy Medical Centre, Khartoum, Sudan | East African medical journal | 82 | 1 | 41548 | 7 |  |  |  |  |  |  | 120 |
| 62 | S. Daradkeh | 2005 | Laparoscopic cholecystectomy: analytical study of 1208 cases | Hepato-Gastroenterology | 52 | 64 | 1011-4 | 7 |  |  |  |  |  |  | 1208 |
| 63 | D. Amott, A. Webb, B. Tulloh | 2005 | Prospective comparison of routine and selective operative cholangiography | ANZ Journal of Surgery | 75 | 6 | 378-382 |  | High | High | Low | Unsure | High | Low | 148 |
| 64 | G. Ersoz, T. Akca, A. Kanik and S. Aydin | 2004 | Efficacy and safety of reuse of disposable laparoscopic instruments in laparoscopic cholecystectomy: a prospective randomized study | Surgical Endoscopy | 18 | 5 | 727-31 |  | Low | Low | Low | Unsure | Low | Low | 62 |
| 65 | I. R. Lai and S. C. Yu | 2004 | Minilaparoscopic (needlescopic) cholecystectomy: a study of 1,011 cases | Surgical Endoscopy | 18 | 10 | 1480-4 | 6 |  |  |  |  |  |  | 1023 |
| 66 | M. Di Paola, M. Catarci, E. Santoro, L. Montemurro, M. Carlini, E. Nanni, L. Alessandroni, R. Angeloni, B. Benini, F. Cristini, A. Dalla Torre, C. De Stefano, A. Gatto, F. Gossetti, S. Manfroni, P. Mascagni, L. Masoni, G. Montalto, D. Polito, E. Puce, G. Silecchia, A. Terenzi, M. Valle, S. Vita and T. Zanarini | 2004 | Bile duct injuries during laparoscopic cholecystectomy: a 1994-2001 audit on 13,718 operations in the area of Rome | Surgical Endoscopy | 18 | 2 | 232-6 | 6 |  |  |  |  |  |  | 6419 |
| 67 | M. Massani, A. Recordare, L. Bonariol, M. Antoniutti, A. Jelmoni and N. Bassi | 2004 | Usefulness of both operative cholangiography and conversion to decrease major bile duct injuries during laparoscopic cholecystectomy | Journal of Hepato-Biliary-Pancreatic Surgery | 11 | 3 | 171-5 | 6 |  |  |  |  |  |  | 1195 |
| 68 | P. T. Antonakis, G. Karatzikos, N. Alexakis and E. Leandros | 2004 | Intraoperative findings and postoperative complications in laparoscopic cholecystectomy: the Greek experience with 5,539 patients in a single center | Journal of Laparoendoscopic & Advanced Surgical Techniques Part A | 14 | 1 | 31-6 | 8 |  |  |  |  |  |  | 5539 |
| 69 | S. C. Guha and S. G. Taylor | 2004 | Laparoscopic cholescystectomy in a rural family practice: the Vivian, LA, experience | Journal of Family Practice | 53 | 3 | 205-8; discussion 209-12 | 6 |  |  |  |  |  |  | 108 |
| 70 | V. Mahatharadol | 2004 | Bile Duct Injuries during Laparoscopic Cholecystectomy: An Audit of 1522 Cases | Hepato Gastroenterology | 51 | 55 | 41974 | 5 |  |  |  |  |  |  | 1522 |
| 71 | S. Panpimanmas | 2004 | Complications of Laparoscopic Cholecystectomy and their Management | Hepato Gastroenterology | 51 | 55 | 42317 | 5 |  |  |  |  |  |  | 348 |
| 72 | K.T. Lee | 2004 | Influence of surgeon volume on clinical and economic outcomes of laparoscopic cholecystectomy | Digestive Surgery | 21 | 42160 | 406-412 | 7 |  |  |  |  |  |  | 916 |
| 73 | S. K. Bhasin | 2004 | Laparoscopic cholecystectomy: An experience of 200 cases | JK Science | 6 | 2 | 73-76 | 5 |  |  |  |  |  |  | 200 |
| 74 | T. Ichihara | 2004 | Tape ligature of cystic duct and fundus-down approach for safety laparoscopic cholecystectomy: Outcome of 500 patients | Hepato Gastroenterology | 51 | 56 | 362-364 | 5 |  |  |  |  |  |  | 505 |
| 75 | H. Y. Docrat, A. A. Haffejee, B. Singh and J. Moodley | 2003 | Cholecystectomy in a predominantly African population before and after the advent of the laparoscopic technique | Surgeon Journal of the Royal Colleges of Surgeons of Edinburgh & Ireland | 1 | 2 | 92-5 | 5 |  |  |  |  |  |  | 156 |
| 76 | L. Fogli, M. Brulatti, S. Boschi, M. Di Domenico, V. Papa and P. Patrizi | 2003 | Conversion rate in laparoscopic cholecystectomy: evolution from 1993 and current state | Journal of Laparoendoscopic & Advanced Surgical Techniques Part A | 13 | 2 | 89-91 | 5 |  |  |  |  |  |  | 1360 |
| 77 | M. A. Zeid, T. Abdallah, A. Fouad, A. A. Eleinien, N. G. el-Hak, G. Eleibiedy, M. A. el-Wahab, A. Sultan, N. Anwar and F. Ezzat | 2003 | Laparoscopic cholecystectomy: a report on 2000 cases | Hepato-Gastroenterology | 50 | 52 | 967-71 | 6 |  |  |  |  |  |  | 2000 |
| 78 | S. Kidney, K. J. Sweeney, A. Zaki, W. A. Tanner and F. V. Keane | 2003 | Changing trends in the management of gallstone disease | Surgical Endoscopy | 17 | 5 | 781-6 | 6 |  |  |  |  |  |  | 152 |
| 79 | M. Barczynski | 2003 | A prospective randomized trial on comparison of low-pressure (LP) and standard-pressure (SP) pneumoperitoneum for laparoscopic cholecystectomy | Surgical Endoscopy and Other Interventional Techniques | 17 | 4 | 533-538 |  | High | Unsure | Unsure | Unsure | High | Low | 74 |
| 80 | Z. Puljiz | 2003 | Bile duct injuries during open and laparoscopic cholecystectomy at Sestre milosrdnice University Hospital from 1995 till 2001 | Acta Clinica Croatica | 42 | 3 | 217-223 | 6 |  |  |  |  |  |  | 2657 |
| 81 | S. Duca | 2003 | Laparoscopic cholecystectomy: Incidents and complications. A retrospective analysis of 9542 consecutive laparoscopic operations | Hpb | 5 | 3 | 152-158 | 5 |  |  |  |  |  |  | 9542 |
| 82 | Luo D | 2003 | Three-dimensional identification of the cystic infundibulum-cystic duct junction: A technique for identification of the cystic duct in laparoscopic cholecystectomy | Hepatobiliary and Pancreatic Diseases International | 2 | 3 | 441-444 | 5 |  |  |  |  |  |  | 12000 |
| 83 | S. Trichak | 2003 | Three-port vs standard four-port laparoscopic cholecystectomy | Surgical Endoscopy | 17 | 9 | 1434-6 | 8 |  |  |  |  |  |  | 100 |
| 84 | D. C. Brooks | 2002 | Contemporary outcomes of ambulatory laparoscopic cholecystectomy in a major teaching hospital | World Journal of Surgery | 26 | 9 | 1117-21 | 7 |  |  |  |  |  |  | 200 |
| 85 | R. Sharma, R. Batra, T. D. Yadav, A. K. Attri and S. P. Kaushik | 2002 | Laparoscopic cholecystectomy: an Indian experience of 1233 cases | Journal of Laparoendoscopic & Advanced Surgical Techniques Part A | 12 | 1 | 42145 | 6 |  |  |  |  |  |  | 1233 |
| 86 | S. V. Shrikhande, V. Rathod, K. Adyanthaya and V. N. Shrikhande | 2002 | Modified technique of gasless laparoscopic cholecystectomy in a developing country: a 5-year experience | Digestive Surgery | 19 | 5 | 366-71; discussion 372 | 8 |  |  |  |  |  |  | 1000 |
| 87 | M. Miroshnik | 2002 | Biliary tract injury in laparoscopic cholecystectomy: Results of a single unit | ANZ Journal of Surgery | 72 | 12 | 867-870 | 6 |  |  |  |  |  |  | 1216 |
| 88 | W. F. Hasaniah | 2002 | Laparoscopic cholecystectomy in 2,750 cases in a teaching hospital in Kuwait | Medical principles and practice : international journal of the Kuwait University, Health Science Centre | 11 | 4 | 176-179 | 6 |  |  |  |  |  |  | 2750 |
| 89 | K. Ludwig | 2002 | Surgical strategies in the laparoscopic therapy of cholecystolithiasis and common duct stones | ANZ Journal of Surgery | 72 | 8 | 547-552 | 6 |  |  |  |  |  |  | 95395 |
| 90 | K. Ludwig | 2002 | Value and consequences of routine intraoperative cholangiography during cholecystectomy | Surgical Laparoscopy, Endoscopy and Percutaneous Techniques | 12 | 3 | 154-159 | 6 |  |  |  |  |  |  | 1332 |
| 91 | G. Sclabas, M. N. Wente, M. Schafer, R. Schlumpf and M. W. Buchler | 2001 | Incidence, risk factors, and prevention of biliary tract injuries during laparoscopic cholecystectomy in Switzerland | World Journal of Surgery | 25 | 10 | 1325-30 | 6 |  |  |  |  |  |  | 12111 |
| 92 | J. J. Reid, M. P. Zahalsky and R. L. Friedman | 2001 | Laparoscopic cholecystectomy in the new millennium | Surgical Endoscopy | 15 | 8 | 867-72 | 6 |  |  |  |  |  |  | 300 |
| 93 | L. Gustafsson, H. Krook, C. E. Nordgren, A. Thorell, G. Wallin and E. Nilsson | 2001 | Laparoscopic cholecystectomy versus mini-laparotomy cholecystectomy: a prospective, randomized, single-blind study | Annals of Surgery | 234 | 6 | 741-9 | 8 |  |  |  |  |  |  | 362 |
| 94 | M. Doganay, M. Dolapci, E. Reis, M. Atli and M. Kologlu | 2001 | Risk factors resulting in conversion of laparoscopic cholecystectomy to open surgery | Surgical Endoscopy | 15 | 9 | 965-8 | 6 |  |  |  |  |  |  | 1000 |
| 95 | W. L. Biffl | 2001 | Routine intraoperative laparoscopic ultrasonography with selective cholangiography reduces bile duct complications during laparoscopic cholecystectomy | Journal of the American College of Surgeons | 193 | 3 | 272-280 | 6 |  |  |  |  |  |  | 594 |
| 96 | D. Maguire, O. J. McAnena and J. Calleary | 2000 | Use of the ultrasonic dissecting scalpel in laparoscopic cholecystectomy | Surgical Endoscopy | 14 | 11 | 1070-3 | 6 |  |  |  |  |  |  | 282 |
| 97 | J. R. Benger | 2000 | Cholecystectomy, conversion and complications | HPB Surgery | 11 | 6 | 373-8 | 4 |  |  |  |  |  |  | 396 |
| 98 | M. Kurose | 2000 | Laparoscopic cholecystectomy: experience with 150 consecutive patients in Kurashiki | Hiroshima Journal of Medical Sciences | 49 | 1 | 42156 | 5 |  |  |  |  |  |  | 150 |
| 99 | S. C. Patel | 2000 | Laparoscopic cholecystectomy at the Aga Khan Hospital, Nairobi | East African medical journal | 77 | 4 | 194-198 | 5 |  |  |  |  |  |  | 135 |
| 100 | S. Lyass | 2000 | Laparoscopic cholecystectomy: What does affect the outcome? A retrospective multifactorial regression analysis | Surgical Endoscopy | 14 | 7 | 661-665 | 5 |  |  |  |  |  |  | 607 |
| 101 | N. Pietra, A. Franze, G. Colla, R. Costi, S. Gobbi and M. Trivelli | 1999 | Routine intravenous cholangiography, selective ERCP, and endoscopic treatment of bile duct stones before laparoscopic cholecystectomy | Gastrointestinal Endoscopy | 50 | 2 | 200-8 | 7 |  |  |  |  |  |  | 1289 |
| 102 | M. T. Cheung | 1999 | Audit of laparoscopic cholecystectomy in a single center | Surgical Laparoscopy and Endoscopy | 9 | 3 | 181-183 | 5 |  |  |  |  |  |  | 1244 |
| 103 | V. K. Thumbe | 1999 | Investigation of bile ducts before laparoscopic cholecystectomy | JSLS : Journal of the Society of Laparoendoscopic Surgeons / Society of Laparoendoscopic Surgeons | 3 | 1 | 23-25 | 6 |  |  |  |  |  |  | 700 |
| 104 | B. D. Matthews | 1999 | Laparoscopic cholecystectomy in an academic hospital: evaluation of changes in perioperative outcomes | JSLS : Journal of the Society of Laparoendoscopic Surgeons / Society of Laparoendoscopic Surgeons | 3 | 1 | 42979 | 6 |  |  |  |  |  |  | 1025 |
| 105 | M. Victorzon | 1999 | Short and long term outcome after laparoscopic cholecystectomy | Annales Chirurgiae et Gynaecologiae | 88 | 4 | 259-263 | 9 |  |  |  |  |  |  | 300 |
| 106 | M. Jabarin, G. Glantzounis, E. T. Lekkas, P. Siakas and S. Stefanaki-Nikou | 1998 | Laparoscopic cholecystectomy using ultrasonically activated coagulating shears | Surgical Laparoscopy & Endoscopy | 8 | 6 | 421-4 |  | High | Unsure | Low | Unsure | High | Unsure | 100 |
| 107 | O. Mjaland, A. Waage, H. Langeggen and J. Holmboe | 1998 | A population-based survey of biliary surgery in Norway. Relationship between patient volume and quality of surgical treatment | Surgical Endoscopy | 12 | 6 | 852-5 | 7 |  |  |  |  |  |  | 4332 |
| 108 | A. J. Sabharwal | 1998 | Laparoscopic cholangiography: A prospective study | British Journal of Surgery | 85 | 5 | 624-626 | 6 |  |  |  |  |  |  | 197 |
| 109 | N. Kurauchi | 1998 | Laparoscopic cholecystectomy: A report on the community hospital experience in Hokkaido | Surgery Today | 28 | 7 | 714-718 | 6 |  |  |  |  |  |  | 1408 |
| 110 | A.H. Kwon | 1998 | Preoperative assessment for laparoscopic cholecystectomy: Feasibility of using spiral computed tomography | Annals of Surgery | 227 | 3 | 351-356 | 6 |  |  |  |  |  |  | 440 |
| 111 | J. S. Wu | 1998 | The evolution and maturation of laparoscopic cholecystectomy in an academic practice | Journal of the American College of Surgeons | 186 | 5 | 554-561 | 6 |  |  |  |  |  |  | 1165 |
| 112 | A. Deleuze, B. de Saxce, C. de Seguin and A. Fingerhut | 1997 | Routine intraoperative cholangiography is feasible and efficient during laparoscopic cholecystectomy | Hepato-Gastroenterology | 44 | 13 | 42207 | 6 |  |  |  |  |  |  | 315 |
| 113 | A. K. Saba and J. L. Peschiera | 1997 | Outpatient laparoscopic cholecystectomy: safe and cost effective? | Surgical Laparoscopy & Endoscopy | 7 | 6 | 487-90 | 5 |  |  |  |  |  |  | 415 |
| 114 | J. T. Ferreira, M. T. Diniz and S. R. Sanches | 1997 | Laparoscopic cholecystectomy in Brazil: analysis of 33,563 cases | International Surgery | 82 | 2 | 208-13 | 7 |  |  |  |  |  |  | 33563 |
| 115 | L. D. Benitez, D. R. Filippone and F. C. Nance | 1997 | Intraoperative cholangiography in laparoscopic cholecystectomy: a review of 734 consecutive cases | American Surgeon | 63 | 2 | 150-6 | 6 |  |  |  |  |  |  | 276 |
| 116 | M. Maciocco, C. Rebuffat, F. Varoli, V. Vergani, G. Rabughino and A. Scarduelli | 1997 | Complications following cholecystectomy | Journal of the Royal College of Surgeons of Edinburgh | 42 | 5 | 324-8 | 5 |  |  |  |  |  |  | 1005 |
| 117 | M. W. Booth, A. Shah, R. A. Pettigrew and J. L. McCall | 1997 | Bile duct imaging and injury: a regional audit of laparoscopic cholecystectomy | Australian & New Zealand Journal of Surgery | 67 | 10 | 706-11 | 6 |  |  |  |  |  |  | 929 |
| 118 | P. C. Sedman, B. M. Jones, C. M. Royston, T. Arulampalam and J. Wellwood | 1997 | Laparoscopic cholecystectomy without operative cholangiogram: 2038 cases over a 5-year period in two district general hospitals | Annals of the Royal College of Surgeons of England | 79 | 5 | 376-80 | 6 |  |  |  |  |  |  | 2038 |
| 119 | A. A. M. Bakr | 1997 | A new modified layout for laparoscopic cholecystectomy | JSLS : Journal of the Society of Laparoendoscopic Surgeons / Society of Laparoendoscopic Surgeons | 1 | 3 | 281-283 | 7 |  |  |  |  |  |  | 119 |
| 120 | M. Ihasz | 1997 | Complications of laparoscopic cholecystectomy in Hungary: A multicentre study of 13833 patients | European Journal of Surgery, Acta Chirurgica | 163 | 4 | 267-274 | 6 |  |  |  |  |  |  | 13833 |
| 121 | D. Collet | 1997 | Laparoscopic cholecystectomy in 1994. Results of a prospective survey conducted by SFCERO on 4,624 cases. Societe Francaise de Chirurgie Endoscopique et Radiologie Operatoire | Surgical Endoscopy | 11 | 1 | 56-63 | 6 |  |  |  |  |  |  | 4624 |
| 122 | S. P. L. Dexter | 1997 | Long operation and the risk of complications from laparoscopic cholecystectomy | British Journal of Surgery | 84 | 4 | 464-466 | 6 |  |  |  |  |  |  | 370 |
| 123 | A. Waage, O. Mjaland and K. Solheim | 1996 | Bile leak after cholecystectomy significance and treatment: results from the National Norwegian Cholecystectomy Registry | International Surgery | 81 | 3 | 276-9 | 6 |  |  |  |  |  |  | 3083 |
| 124 | D. Velnic, B. Cvitanovic, Z. Rasic and Z. Perko | 1996 | Laparoscopic cholecystectomy: results after 1000 procedures | Acta Medica Croatica | 50 | 3 | 147-9 | 6 |  |  |  |  |  |  | 1000 |
| 125 | E. Eypasch, R. Lefering, A. Paul, E. Neugebauer and H. Troidl | 1996 | Laparoscopic cholecystectomy for acute cholecystitis: is it really safe? | World Journal of Surgery | 20 | 1 | 43-8; discussion 48-9 | 9 |  |  |  |  |  |  | 54 |
| 126 | G. Bell and G. M. Fullarton | 1996 | Incidence and nature of bile duct injuries following laparoscopic cholecystectomy: an audit of 5913 cases. West of Scotland Laparoscopic Cholecystectomy Audit Group | British Journal of Surgery | 83 | 10 | 1356-60 | 6 |  |  |  |  |  |  | 5913 |
| 127 | I. Airo, C. Haglund, E. Kivilaakso, T. Kiviluoto, J. Palm, M. Paakkonen, S. Ristkari and K. V. Smitten | 1996 | Laparoscopic cholecystectomy: the Finnish experience | Annales Chirurgiae et Gynaecologiae | 85 | 3 | 208-11 | 6 |  |  |  |  |  |  | 5742 |
| 128 | K. H. Kwong, W. Y. Lau, S. C. Chung and A. K. Li | 1996 | Absorbable clips for cystic duct ligation in laparoscopic cholecystectomy | Surgical Endoscopy | 10 | 1 | 49-51 | 6 |  |  |  |  |  |  | 297 |
| 129 | M. M. Thombare, S. S. Sikora, R. Saxena, V. K. Kapoor and S. P. Kaushik | 1996 | Morbidity and mortality of laparoscopic cholecystectomy in an institutional setup | Journal of Laparoendoscopic Surgery | 6 | 6 | 393-7 | 6 |  |  |  |  |  |  | 433 |
| 130 | E. Leandros, N. Dourakis, C. Birbas, G. Delibaltadakis and B. Golematis | 1995 | Laparoscopic cholecystectomy. Intraoperative findings and postoperative complications | Surgical Endoscopy | 9 | 8 | 889-93 | 6 |  |  |  |  |  |  | 1788 |
| 131 | T. Pozarliev and G. T. Todorov | 1995 | Laparoscopic cholecystectomy: 700 consecutive cases | International Surgery | 80 | 4 | 296-8 | 6 |  |  |  |  |  |  | 700 |
| 132 | J. W. Lorimer | 1995 | Intraoperative cholangiography is not essential to avoid duct injuries laparoscopic cholecystectomy | American Journal of Surgery | 169 | 3 | 344-347 | 5 |  |  |  |  |  |  | 525 |
| 133 | O. N. M. Panton | 1995 | Laparoscopic cholecystectomy: A continuing plea for routine cholangiography | Surgical Laparoscopy and Endoscopy | 5 | 1 | 43-49 | 6 |  |  |  |  |  |  | 236 |
| 134 | Wherry DC | 1994 | An external audit of laparoscopic cholecystectomy performed in medical treatment facilities of the department of Defense | Annals of Surgery | 220 | 5 | 626-634 |  |  |  |  |  |  |  | 5607 |
| 135 | Schlumpf R | 1994 | A nation's experience in laparoscopic cholecystectomy. Prospective multicenter analysis of 3722 cases | Surgical Endoscopy | 8 | 1 | 35-41 |  |  |  |  |  |  |  | 3722 |
| 136 | Dunn D | 1994 | Laparoscopic cholecystectomy in England and Wales: results of an audit by the Royal College of Surgeons of England | Annals of the Royal College of Surgeons of England | 76 | 4 | 269-275 | 6 |  |  |  |  |  |  | 8035 |
| 137 | Birdi I | 1994 | Laparoscopic cholecystectomy in Leicester: An audit of 555 patients | Annals of the Royal College of Surgeons of England | 76 | 6 | 390-395 |  |  |  |  |  |  |  | 555 |
| 138 | Cox MR | 1994 | Minimizing the risk of bile duct injury at laparoscopic cholecystectomy | World Journal of Surgery | 18 | 3 | 422-427 |  |  |  |  |  |  |  | 410 |
| 139 | Soper NJ | 1993 | Laparoscopic cholecystectomy: experience of a single surgeon | World Journal of Surgery | 17 | 1 | 16-20 |  |  |  |  |  |  |  | 415 |
| 140 | Orlando R 3rd | 1993 | Laparoscopic cholecystectomy. A statewide experience. The Connecticut Laparoscopic Cholecystectomy Registry | Archives of Surgery | 128 | 5 | 494-499 |  |  |  |  |  |  |  | 4640 |
| 141 | Collet D | 1993 | Conversions and complications of laparoscopic cholecystectomy. Results of a survey conducted by the French Society of Endoscopic Surgery and Interventional Radiology | Surgical Endoscopy | 7 | 4 | 334-338 |  |  |  |  |  |  |  | 2955 |
| 142 | Perissat J | 1992 | Laparoscopic cholecystectomy: the state of the art. A report on 700 consecutive cases | World Journal of Surgery | 16 | 6 | 1074-1082 |  |  |  |  |  |  |  | 700 |
| 143 | Martin IG | 1992 | Laparoscopic cholecystectomy as a routine procedure for gallstones: results of an 'all-comers' policy | British Journal of Surgery | 79 | 8 | 807-810 |  |  |  |  |  |  |  | 162 |
| 144 | Smith EB | 1992 | Complications of laparoscopic cholecystectomy | Journal of the National Medical Association | 84 | 10 | 880-882 |  |  |  |  |  |  |  | 1009 |
| 145 | Larson GM | 1992 | Multipractice analysis of laparoscopic cholecystectomy in 1,983 patients | American Journal of Surgery | 163 | 2 | 221-226 |  |  |  |  |  |  |  | 1983 |
| 146 | Bailey RW | 1991 | Laparoscopic cholecystectomy. Experience with 375 consecutive patients | Annals of Surgery | 214 | 4 | 531-541 |  |  |  |  |  |  |  | 375 |
| 147 | Wilson P | 1991 | Elective laparoscopic cholecystectomy for "all-comers" | Lancet | 338 | 8770 | 795-797 |  |  |  |  |  |  |  | 180 |
| 148 | Southern Surgeons Club | 1991 | A prospective analysis of 1518 laparoscopic cholecystectomies. The Southern Surgeons Club.[Erratum appears in N Engl J Med 1991 Nov 21;325(21):1517-8] | New England Journal of Medicine | 324 | 16 | 1073-1078 |  |  |  |  |  |  |  | 1518 |
| 149 | Bucher P | 2011 | Randomized clinical trial of laparoendoscopic single-site versus conventional laparoscopic cholecystectomy | British Journal of Surgery | 98 | 12 | 1695-1702 |  | low | high | high | high | unsure | low | 75 |
| 150 | Wallace DH | 1997 | Effect of a no-conversion policy on patient outcome following laparoscopic cholecystectomy | British Journal of Surgery | 84 | 12 | 1680-1682 | 6 |  |  |  |  |  |  | 127 |
| 151 | Poon CM | 2003 | Two-port vs four-port laparoscopic cholecystectomy: A prospective randomized controlled trial | Surgical Endoscopy and Other Interventional Techniques | 17 | 10 | 1624-1627 |  | low | low | low | low | unsure | unsure | 58 |
