# Supplementary material for: Outcome trends and safety measures after 30 years of laparoscopic cholecystectomy: a systematic review and pooled data analysis
Source: Surg Endosc. 2018 Mar 19;32(5):2175–83. doi: 10.1007/s00464-017-5974-2 (PMC5897463; doi:10.1007/s00464-017-5974-2)

**Appendix 2. Meta-analysis**

Forest plot of studies comparing bile duct injury rates for interventions as shown. SILS = Single incision laparoscopic surgery, CLC = conventional laparoscopic cholecystectomy, IOC = intraoperative cholangiogram


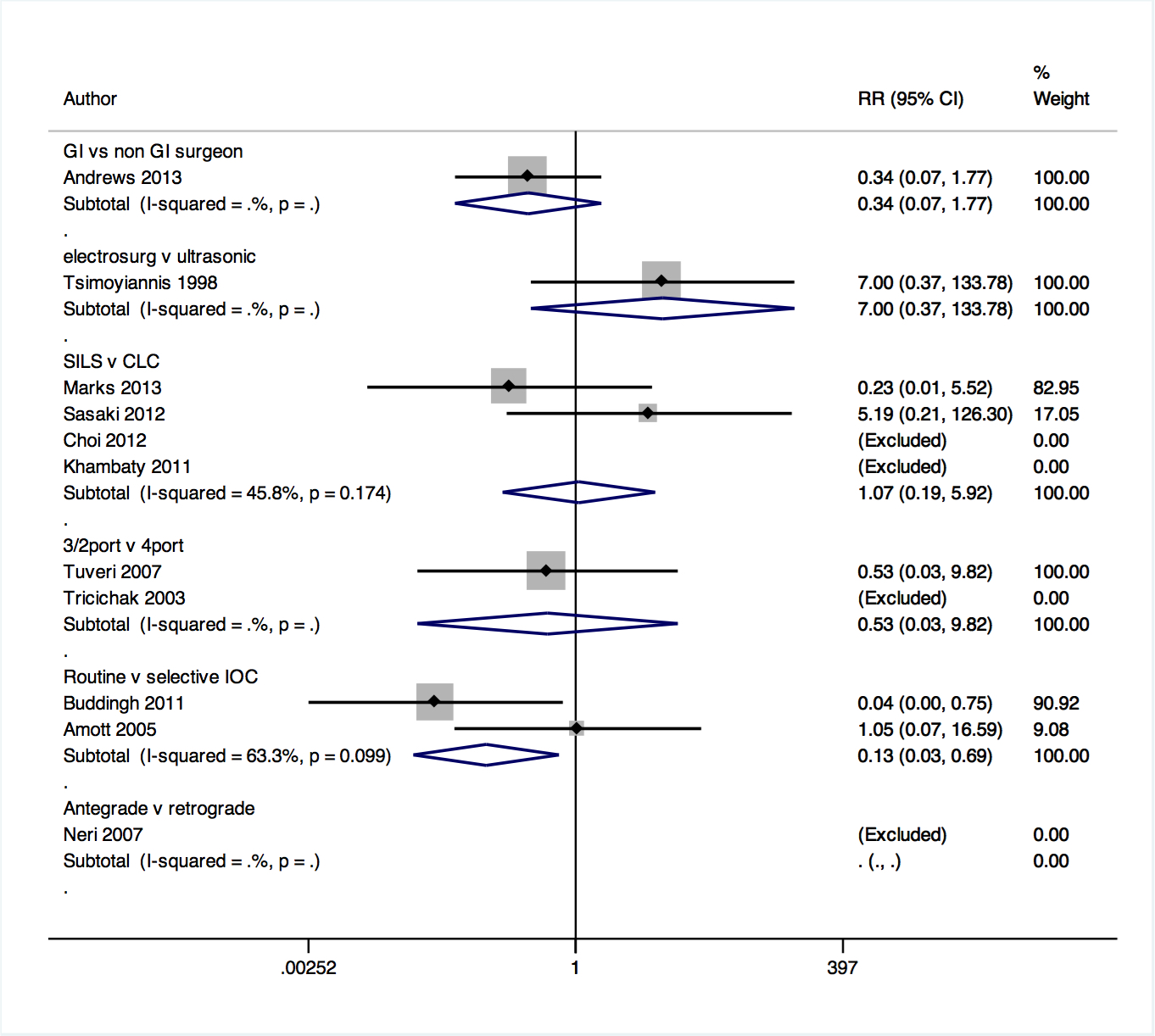

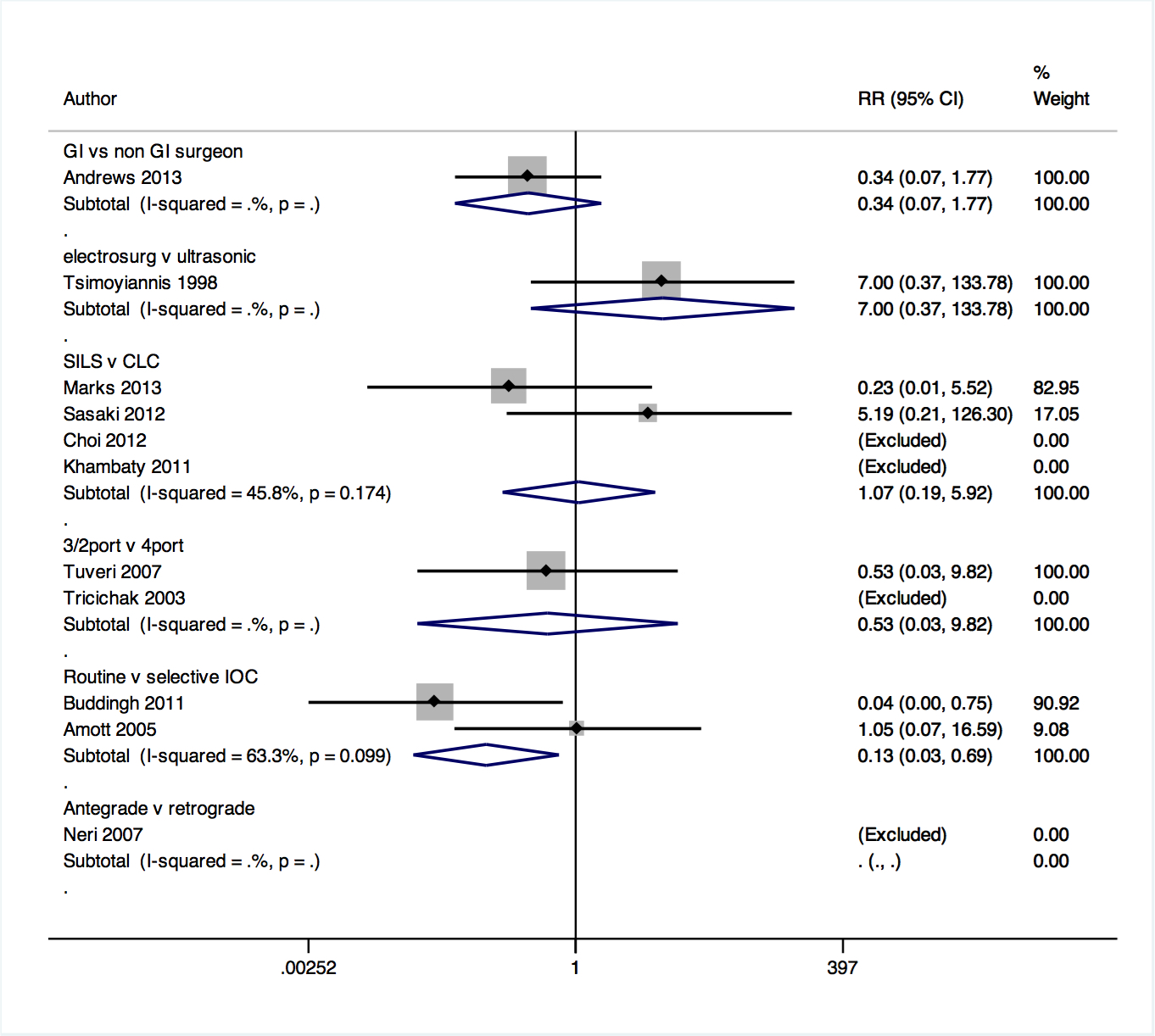

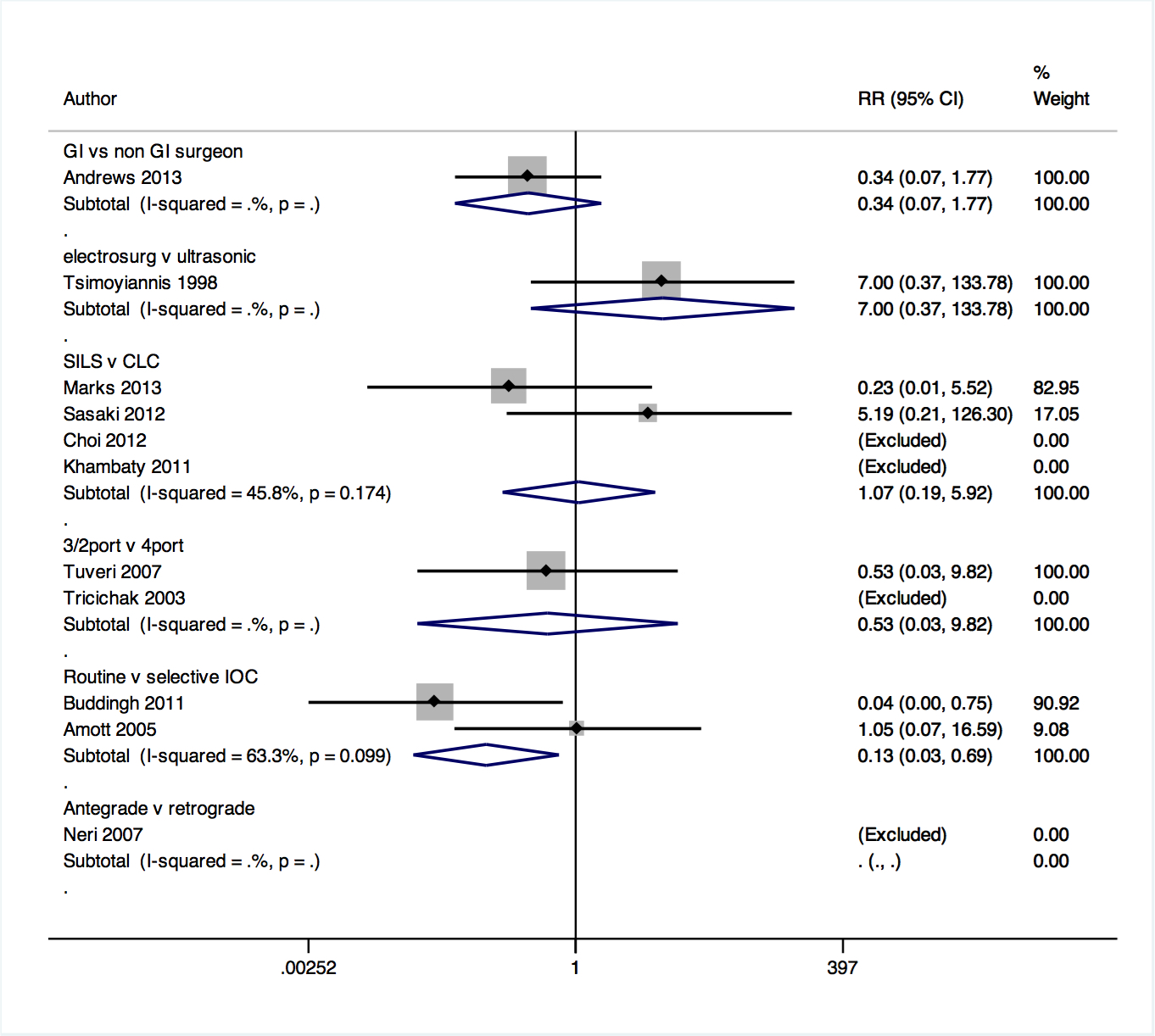

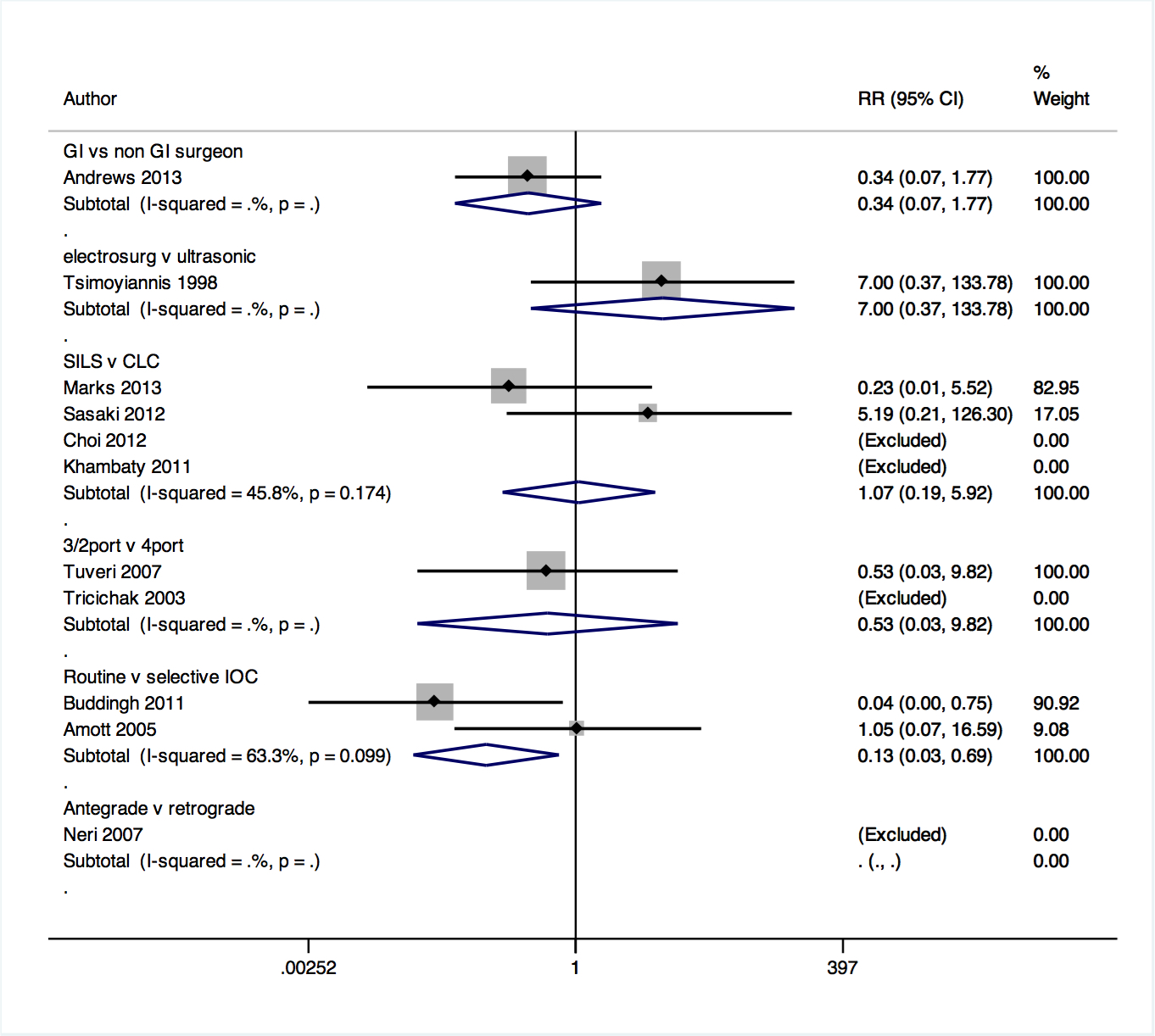

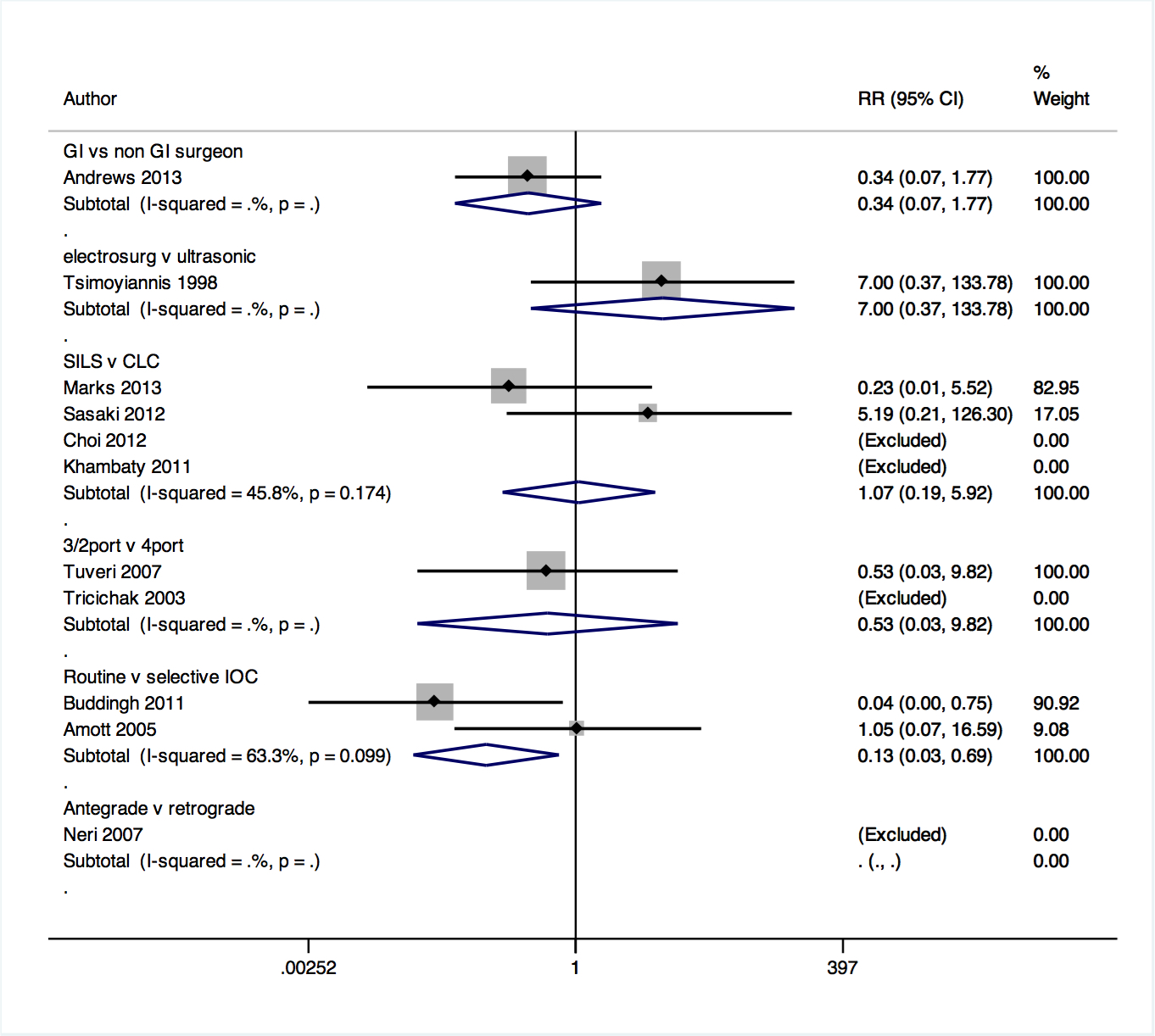


Forest plot of studies comparing bile duct injury rates for interventions as shown. Electrosurg = electrosurgical dissection instrument, ultrasonic = ultrasonic dissection instrument, SILS = Single incision laparoscopic surgery, CLC = conventional laparoscopic cholecystectomy, IOC = intraoperative cholangiogram


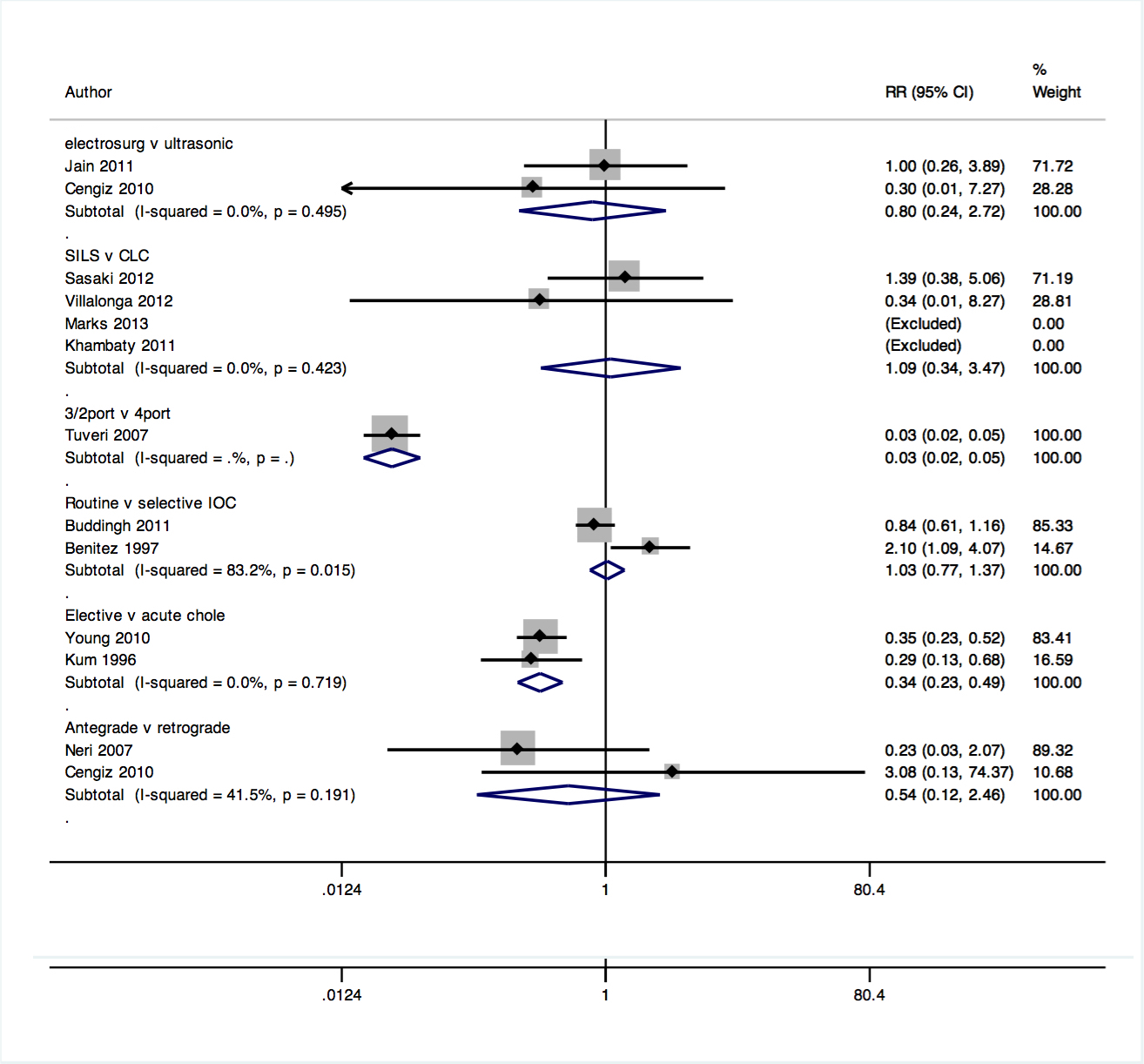

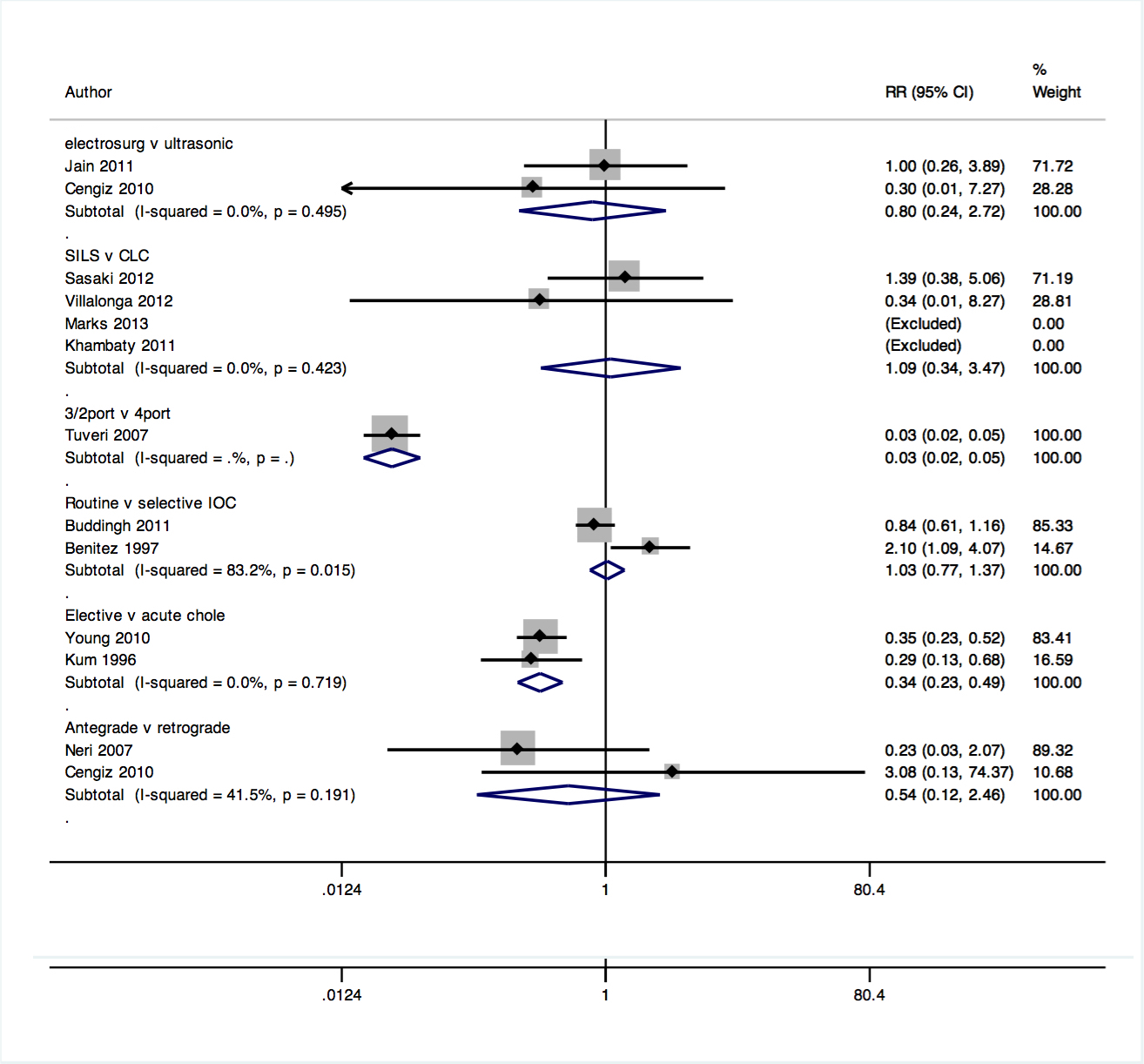

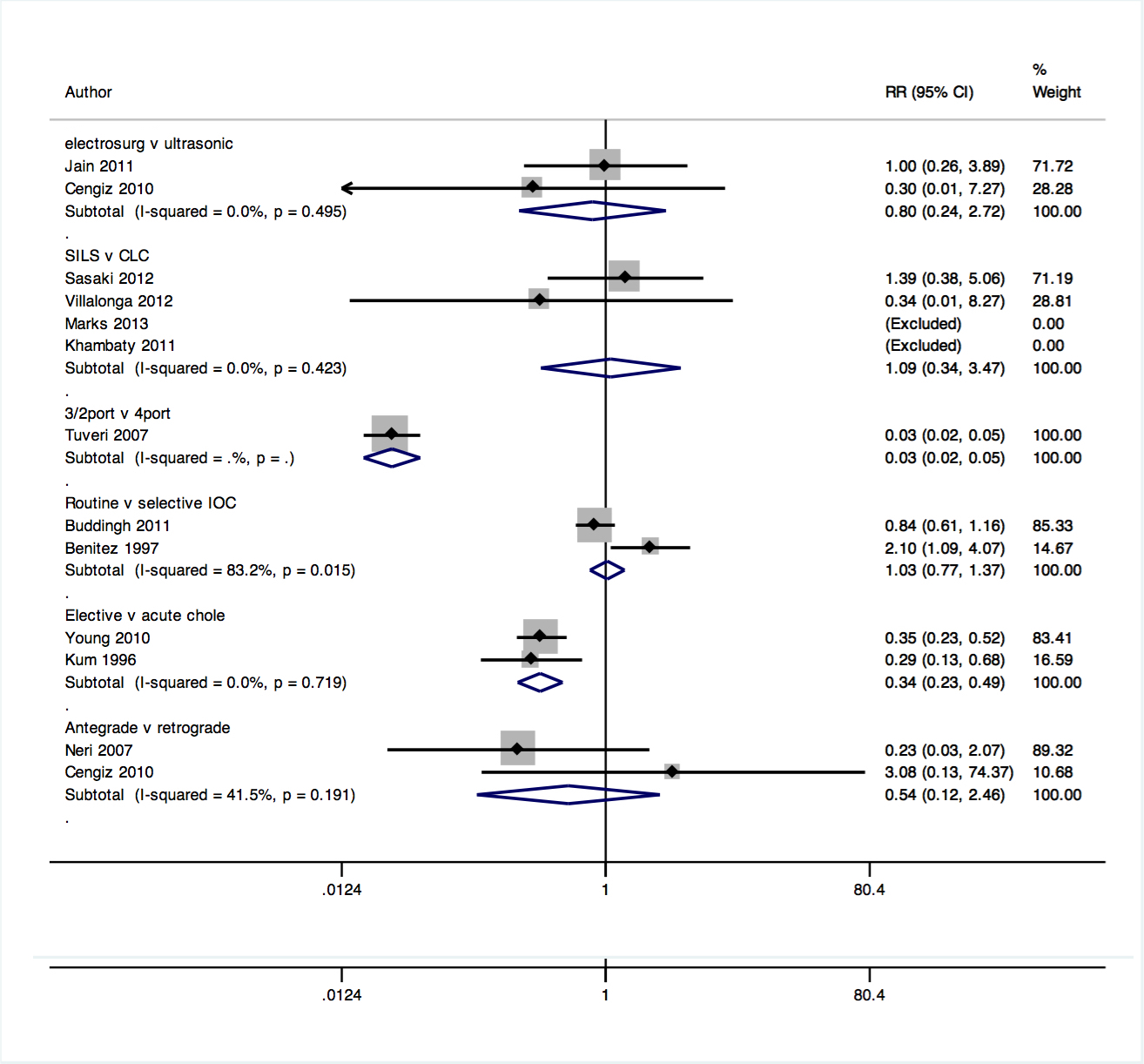

Supplement: Supplementary file 2 — Supplementary material 2 (DOCX 8089 KB) [file 464_2017_5974_MOESM2_ESM.docx]
